# Using a Supramolecular Monomer Formulation Approach to Engineer Modular, Dynamic Microgels, and Composite Macrogels

**DOI:** 10.1002/adma.202405868

**Published:** 2024-10-27

**Authors:** Maritza M. Rovers, Theodora Rogkoti, Bram K. Bakker, Kalpit J. Bakal, Marcel H.P. van Genderen, Manuel Salmeron‐Sanchez, Patricia Y.W. Dankers

**Affiliations:** ^1^ Institute for Complex Molecular Systems Eindhoven University of Technology P.O. Box 513 Eindhoven 5600 MB The Netherlands; ^2^ Department of Biomedical Engineering Laboratory of Chemical Biology Eindhoven University of Technology P.O. Box 513 Eindhoven 5600 MB The Netherlands; ^3^ Centre for the Cellular Microenvironment University of Glasgow, Advanced Research Centre 11 Chapel Lane Glasgow G11 6EW UK; ^4^ Department of Mechanical Engineering Eindhoven University of Technology P.O. Box 513 Eindhoven 5600 MB The Netherlands; ^5^ Institute for Bioengineering of Catalonia (IBEC) The Barcelona Institute for Science and Technology (BIST) Barcelona 08028 Spain; ^6^ Institució Catalana de Recerca i Estudis Avançats (ICREA) Barcelona 08010 Spain; ^7^ Department of Chemical Engineering and Chemistry Eindhoven University of Technology P.O. Box 513 Eindhoven 5600 MB The Netherlands

**Keywords:** cell culture, droplet‐based microfluidics, hydrogel, microgel, multiscale modularity, supramolecular biomaterial, synthetic extracellular matrix

## Abstract

Microgels show advantages over bulk hydrogels due to convenient control over microgel size and composition, and the ability to use microgels to modularly construct larger hierarchical scaffold hydrogel materials. Here, supramolecular chemistry is used to formulate supramolecular polymer, dynamic microgels solely held together by non‐covalent interactions. Four‐fold hydrogen bonding ureido‐pyrimidinone (UPy) monomers with different functionalities are applied to precisely tune microgel properties in a modular way, via variations in monomer concentration, bifunctional crosslinker ratio, and the incorporation of supramolecular dyes and peptides. Functionalization with a bioactive supramolecular cell‐adhesive peptide induced selectivity of cells toward the bioactive microgels over non‐active, non‐functionalized versions. Importantly, the supramolecular microgels can also be applied as microscale building blocks into supramolecular bulk macrogels with tunable dynamic behavior: a robust and weak macrogel, where the micro‐ and macrogels are composed of similar molecular building blocks. In a robust macrogel, microgels act as modular micro‐building blocks, introducing multi‐compartmentalization, while in a weak macrogel, microgels reinforce and enhance mechanical properties. This work demonstrates the potential to modularly engineer higher‐length‐scale structures using small molecule supramolecular monomers, wherein microgels serve as versatile and modular micro‐building units.

## Graphical abstract

Microgels with engineerable properties are created using supramolecular monomers. The inherent modularity offers versatility, allowing inclusion of functionalities, e.g. dyes and cell adhesive additives, providing control over microgel composition. Integrated into robust and dynamic macrogels, microgels act as modular micro‐building blocks, introducing multi‐compartmentalization and reinforcing mechanical properties. This modular control spans across the nano, micro, and macro length scale.

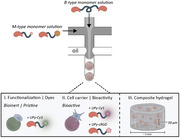

## 1 Introduction

Hydrogels, either natural or synthetic, have been extensively studied as biomaterials to mimic the extracellular matrix (ECM) as a structural support to cells to promote tissue regeneration.^[^ ^1^ ^]^ Nonetheless, hydrogels often exhibit isotropic characteristics, along with a nanoporous mesh that complicates the infiltration of cells, while naturally the ECM displays a heterogeneous multiscale complexity at a nano, micro, and macrolength scales.^[^ ^3^ ^]^ Recently, attention has been directed toward novel approaches for miniaturizing traditional bulk hydrogels to the microscale, termed microgels. The unique microscale geometry, with their increased surface area‐to‐volume ratio, promotes efficient nutrient and waste exchange, consequently stimulating more dynamic cellular processes and enhancing interactions among cells and biomaterials.^[^ ^4^ ^]^ Additionally, the relative small size allows for minimally invasive injection of microgels or cell‐laden microgels as delivery vehicle.^[^ ^5^, ^6^ ^]^

In the field of tissue engineering, microgels can be integrated into traditional macroscale bulk hydrogels to serve as microsized building blocks with a defined molecular composition. This allows individual spatial control over the micro‐ and macro‐environment to more precisely reflect the structural and compositional complexity of living tissue.^[^ ^7^ ^]^ This is relevant since naturally the body's biological system also possesses complex architectures featuring multiple compartments prevalent in nearly every organ.^[^ ^8^ ^]^ The Werner lab already proved that microgels when mixed into macrogels could potentially serve as such microarchitectures with defined function.^[^ ^9^ ^]^ Moreover, this concept was used for bioprinting applications by formulating modular bioinks containing (cells encapsulated in) microgels in an injectable bulk gel to create hydrogels with distinct micro‐ and macro‐environmental properties.^[^ ^7^, ^10^ ^]^ This approach not only allows the incorporation of various functional domains in a bulk hydrogel, but has also shown the potential to provide mechanical toughness to the macrogel, these so called microgel‐reinforced hydrogels.^[^ ^11^, ^12^ ^]^

Alternatively, microgels can be assembled into larger (heterogeneous) micro‐engineered scaffolds with sizes ranging from several micrometers to millimeters with or without the use of another matrix or macrogel. These so‐called granular hydrogels are formed through a bottom‐up approach via physical reactions, external driving forces, or cell‐cell and cell‐material interactions.^[^ ^13^, ^14^, ^15^, ^16^, ^17^ ^]^ Many labs have demonstrated the benefits of microgel assembly, leading to the generation of void spaces, giving rise to microporous scaffolds that promote cellular growth and tissue formation. For example, the Segura lab developed microporous annealed particle (MAP) scaffolds through a thiol‐norbornene click reaction that anneals hyaluronic acid microgels together that support cellular growth.^[^ ^18^ ^]^ Additionally, the Leijten lab engineered stimuli‐responsive and cell‐adhesive micromaterials (SCMs) consisting of dextran‐based microgels. This assembly was facilitated by the dextran backbone modification with tyramine and biotin moieties, enabling in‐situ crosslinking, post‐curing, and post‐functionalization to facilitate cell adhesion.^[^ ^19^ ^]^ The Pich and Laporte lab created cell‐induced interlinked scaffolds by combining cells with bio‐based dextran microgels, inducing the formation of the MAP scaffold through cellular self‐organization.^[^ ^20^ ^]^ In these studies, the granular scaffolds were composed of hybrid microgels based on modified biopolymers. However, often additional modifications are required to facilitate microgel formation, or to render them bioactive and suitable to cell growth.^[^ ^21^ ^]^ Moreover, the utilization of solely natural building blocks restricts the tunability of the microgel components, such as stiffness, porosity, and degradability, often being intrinsic to the employed macromolecular building blocks. Consequently, polymer blends or composites have emerged, however issues such as phase separation may occur.^[^ ^22^ ^]^

The formulation of microgels with distinct desired properties could be facilitated by using purely synthetic building blocks, as these can be tailored for specific applications and can offer biochemical cues for cell culture by the incorporation of bioactive additives.^[^ ^23^ ^]^ For example, the Lutolf group showed that a bioactive microgel library could be generated with hugely diverse compositions without the requirement of post‐modification steps. This was achieved by using synthetic branched poly(ethylene glycol) (PEG) based building blocks crosslinked by stepwise copolymerization via a Michael‐type addition reaction.^[^ ^24^ ^]^ Also, the Anseth group formulated PEG microgels functionalized with a dibenzocyclooctyne (DBCO) or azide to assemble the microgels into a microporous scaffold.^[^ ^25^, ^26^ ^]^ Microgels were functionalized with the cellular adhesive arginine‐glycine‐aspartate (RGD) peptide to allow cell culture of human mesenchymal stem cells (hMSCs). These synthetic microgels are typically constructed through controlled polymerization or cross‐linking processes based on covalent chemistry. From bulk hydrogels it is known that this often associated with stable structures with limited adaptability, responsiveness to external stimuli, and lacking intrinsic dynamic properties.^[^ ^27^ ^]^

In this respect, supramolecular chemistry emerged as a promising system to formulate hydrogels. The self‐complementary supramolecular building blocks exhibit the capability to specifically interact through non‐covalent interactions such as hydrogen bonding, hydrophobic interactions, and π‐π interactions.^[^ ^28^ ^]^ This gives unique material properties in terms of dynamics, adaptability, processibility, and control over their mechanical and bioactive properties.^[^ ^29^ ^]^ Several research groups have already shown the promising applications of supramolecular bulk hydrogels as ECM mimics, featuring benzene‐1,3,5‐tricarboxamide (BTA), bis‐urea (BU) and ureido‐pyrimidinone (UPy) moieties, self‐assembling ultrashort peptides (SUPs), and peptide amphiphiles (PA).^[^ ^30^, ^31^, ^32^, ^33^, ^34^ ^]^ Recently, this field has made the transition toward the formulation of supramolecular microgels. For example, the Pich group fabricated pH‐degradable supramolecular microgels using amphiphilic stimuli‐responsive polymers (PVCL) and a natural polyphenol (TA) as building blocks.^[^ ^35^ ^]^ Furthermore, they developed electroactive and degradable supramolecular microgels based on biomacromolecular building blocks, which enables the controlled release of therapeutic drugs.^[^ ^36^ ^]^ In the Seiffert group, microgels were fabricated from PEG precursors functionalized with bipyridine (bpy) moieties. These complex to iron(II), which leads to polymer–polymer linkage and the formation of a purely supramolecular microgels.^[^ ^37^ ^]^ Lastly, the Hauser group constructed a microcarrier cell delivery system based on SUPs of tri‐ to hexapeptides with a characteristic amphiphilic motif of a hydrophobic tail capped by a polar head that form ECM mimicking bundles.^[^^]^ They used a modular tissue‐engineering approach by embedding the microgels with endothelial cells into a macro‐hydrogel containing fibroblastic cells. This modularity, although a common and beneficial aspect, is not an inherent feature across all supramolecular interactions or structures by definition.

Modularity in supramolecular microgels refers to the ability to design and assemble these microgels from discrete, customizable building blocks, allowing for a high degree of control over their structure, properties, and functionality.^[^ ^38^, ^39^ ^]^ The concept of modularity is crucial in the context of supramolecular microgels for several reasons: 1) tailored properties by selecting and combining different building blocks, 2) multifunctionality by incorporating multiple functionalities within a single microgel, and 3) adaptability and versatility by the reversible nature of non‐covalent interactions that allow for dynamic rearrangements. Moreover, employing modular molecular building blocks for microgel formulation facilitates their utilization in a modular tissue engineering approach, allowing for the bottom‐up formation of tissues from these microgels.

Owing to the beneficial role of modularity in microgels, we here aim to engineer ureido‐pyrimidinone (UPy) monomers into freestanding modular supramolecular polymer microgels held together exclusively by non‐covalent interactions using a water‐in‐oil‐emulsion microfluidic setup.^[^ ^40^ ^]^ As base supramolecular hydrogel forming system we used our monofunctional (M‐type) UPy‐monomers and bifunctional (B‐type) crosslinkers.^[^ ^29^ ^]^ These UPy‐moieties are linked to an alkyl spacer to shield the hydrogen bonds in water, creating a hydrophobic pocket when UPy‐dimers assemble into 1D stacks. The modularity of the UPy‐monomers enables copolymerization with bioactive UPy‐monomer additives within the nanoscopic supramolecular fiber, such as UPy‐cRGD or UPy‐functionalized dyes, allowing for customization of microgel properties, including w/v% variations by adjusting UPy‐concentration or modulate molecular exchange by altering the ratio between B‐ and M‐type monomers.^[^ ^29^, ^33^, ^41^, ^42^, ^43^, ^44^, ^45^ ^]^ Lastly, we show how to apply these supramolecular microgels as modular micro‐building blocks to create composite hydrogels at a macroscale.

## 2 Results and Discussion

### 2.1 Microgel Formulation based on UPy‐Monomers

**Figure 1:**
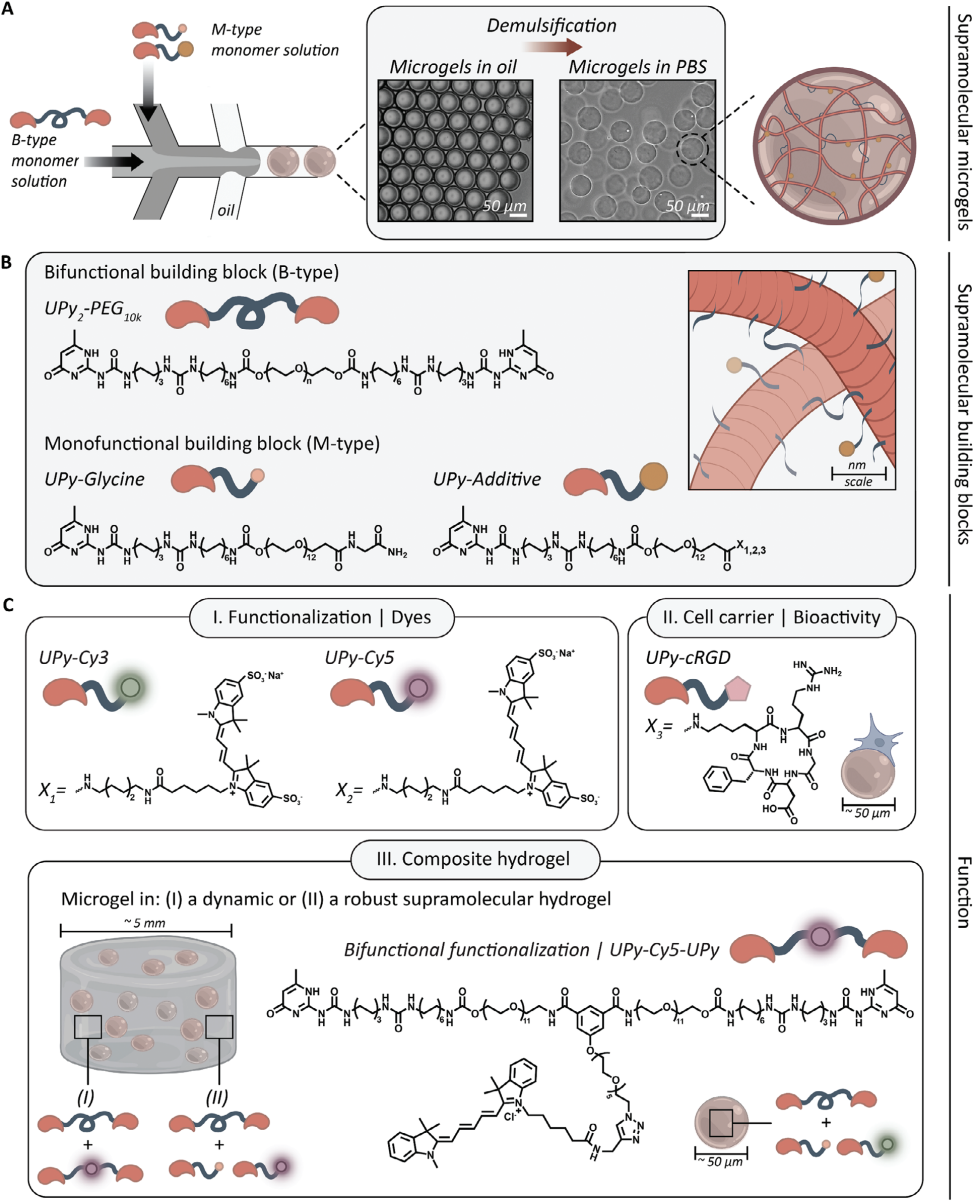
Aim and molecular design of this study. A) Droplet‐based microfluidic formulation of supramolecular microgel building blocks, including brightfield microscopy image of 2.0 w/v% microgels in PBS after demulsification. All scale bars represent 50 µm. B) Supramolecular building blocks used in this study: bifunctional UPy_2_‐PEG_10kDa_ (B‐type; n ≈ 227) and monofunctional UPy‐glycine (M‐type) including UPy‐moiety with variable end group X_1‐3_. C) The systems modularity allows for microgel functionalization by means of I. dyes (UPy‐Cy3 or UPy‐Cy5) or II. bioactivity through the incorporation of UPy‐functionalized additives, such as UPy‐cRGD, facilitating cellular adhesion. III. The microgels can be used as modular micro‐building blocks to create composites at a macroscale. Where this macrogel is either a (I) dynamic or (II) robust supramolecular hydrogel, that can be functionalized with a UPy‐Cy5 or UPy‐Cy5‐UPy additive.

Supramolecular microgels were formulated using two distinct types of supramolecular building blocks: the M‐type UPy‐Glycine monomer and the B‐type bifunctional UPy_2_‐PEG_10kDa_ crosslinker (**Figure** **1**).^[^ ^29^ ^]^ The utilization of these supramolecular building blocks to create microgels entails several essential prerequisites: 1) it is crucial that the mixing between the building blocks from the M‐type and B‐type pre‐solutions occurs rapidly to prevent channel clogging and under controlled conditions of equal flow, while 2) the stability of the formed microgel is sufficiently high to prevent their dissolution or amalgamation into a bulk gel. Each of the building blocks was dissolved separately at predefined concentrations in aqueous solution and drawn separately into a pipetted‐tip based droplet microfluidic setup. Within this setup, the B‐type solution was directed to flow from the middle inlet, while the M‐type solution was loaded in the inner inlet (Figure S1A, Supporting Information). At the point of intersection, both hydrogelators mix in a 1:1 volume ratio where the faster oil phase shears microdroplets from the aqueous polymer stream. In this droplet, it proposed that the M‐type monomers form fibrous structures, while the B‐type molecules crosslink these into a transient network via supramolecular interactions. As this gelation occurs in oil, the influence of evaporation is eliminated which ensures that all microgels gelate under the same conditions. After proper gelation of at least 2 h at 37 °C, we showed that the supramolecular microgels exhibited sufficient stability, enabling their isolation from the oil phase through demulsification (Figure 1A; Figure S1B,C, Supporting Information). Homogeneous co‐encapsulation of UPy‐functionalized monomer additives in the microgel was easily accomplished on chip by introducing the additive into the hydrogel presolution, exemplified by the even distribution of the UPy‐Cy3 dye in the microgel (Figure S1D, Supporting Information).

### 2.2 Modularly Tuning Microgel Composition

The modularity of the UPy‐system offers the versatility to create diverse microgel compositions.

**Figure 2:**
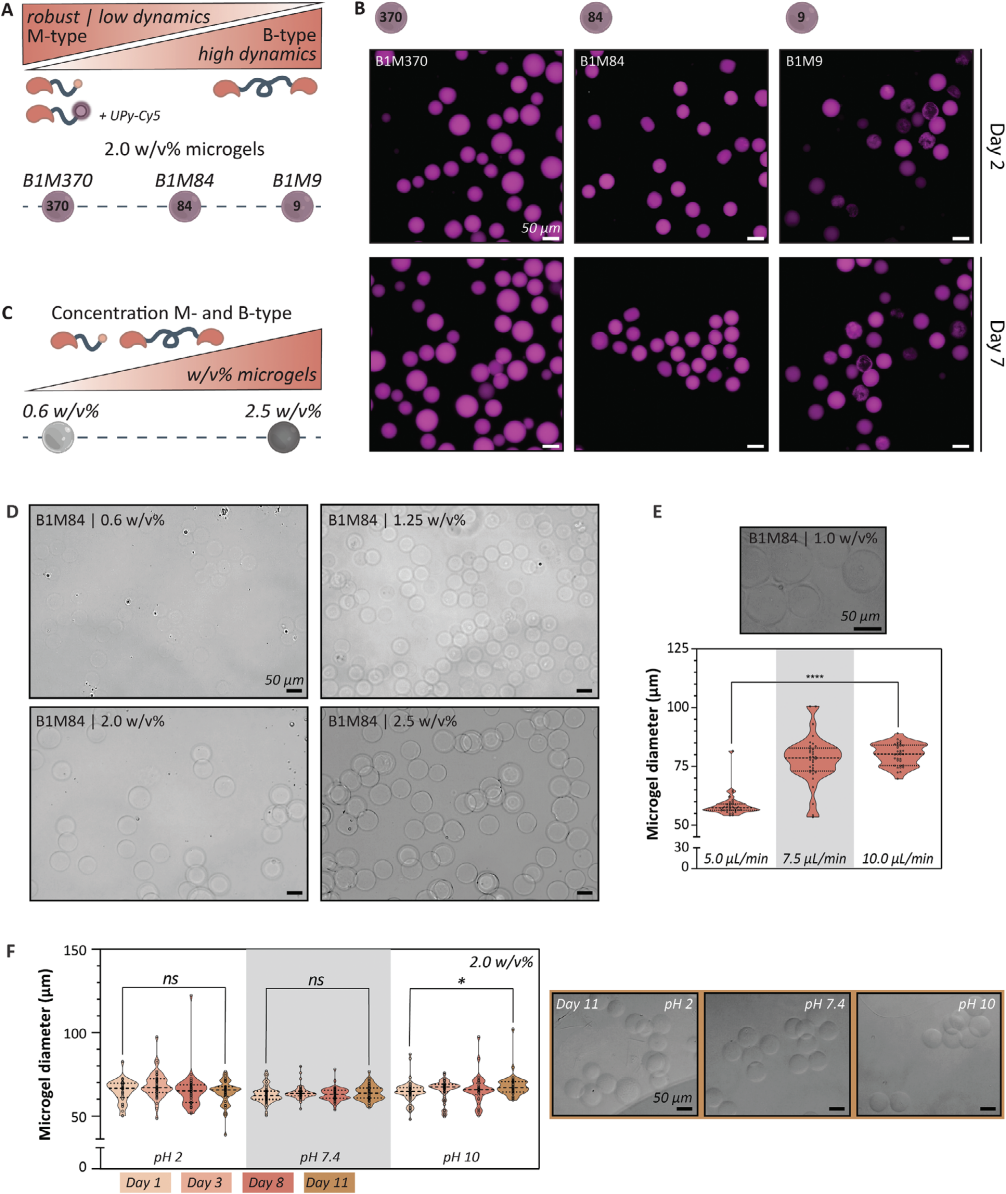
The modularity of the UPy‐system offers the versatility to create diverse microgel compositions. A) Tuning the molecular exchange dynamic by altering the molar ratio between B‐ and M‐type monomers in the microgel. Microgels with a fluorescent UPy‐Cy5 dye were formulated with the following molar ratios B‐ to M‐type monomers: B1M370, B1M84, and B1M9. B) All formulations (B1M370, B1M84, and B1M9) resulted in the formation of microgels that are stable and do not dissolute or amalgamate into a bulk gel after 7 days. C‐D) UPy‐based microgels can be formulated at various concentrations (i.e., w/v%): 0.6, 1.25, 2.0, and 2.5 w/v% microgels. E) By controlling the flow speed (5.0, 7.5, and 10.0 µL min^−1^) of the B‐type and M‐type hydrogel presolutions, different microgel sizes could be obtained (Kolmogorov‐Smirnov test; ****p < 0.0001; *n* = 34 microgels; values shown as mean ± SD). F) The microgels are considered stable by retaining their size at basic, acidic, and physiological conditions. Only microgels stored for a period of 11 days showed a slight decrease in diameter (Kolmogorov‐Smirnov test; *p < 0.05; *n* = between 32 and 60 microgels; values shown as mean ± SD). All scale bars represent 50 µm.

Diba et al. showed that an increased M‐type relative to B‐type molar ratio resulted in hydrogels with slower stress relaxation, which was attributed to a reduced dynamic behavior of the hydrogel network.^[^ ^29^ ^]^ Here we demonstrated the formulation of microgels with tunable molecular exchange dynamics by altering the molar ratio between B‐ and M‐type monomers in the microgel (**Figure** **2**A). To this end, 2.0 w/v% microgels with a fluorescent UPy‐Cy5 dye were formulated with the following molar ratios B‐ to M‐type monomers: B1M370, B1M84, and B1M9 (Table S1, Supporting Information). All formulations resulted in the formation of microgels that are stable and do not dissolute or amalgamate into a bulk gel after 7 days (Figure 2B). For all further experiments, microgels with a B1M84 ratio were used, known for their robustness and slow relaxation previously studied in bulk hydrogels.^[^ ^29^ ^]^

Furthermore, we demonstrated the formulation of microgels at various polymer concentration (i.e., w/v% of the microgel) by adjusting the concentrations of the microgel pre‐solutions, while maintaining a fixed molar ratio between the M‐type and B‐type building blocks (Figure 2C,D; Table S2, Supporting Information). To this end, 0.6, 1.25, 2.0, and 2.5 w/v% microgels were formulated. Notably, microgels at 0.6 w/v% exhibited proper gelation and could be isolated from the oil phase, while microgel pre‐solutions aimed at producing 2.5 w/v% microgels maintained the capability to flow through the channels.

In addition, microgel size could be controlled by adjusting the microfluidic flow speed of the hydrogelator solutions.^[^ ^46^ ^]^ For this, 1.0 w/v% microgels were chosen as it fell within the range of the tested conditions (0.6–2.5 w/v% microgels). They were formulated under different flow speeds, which resulted in an increase in microgel diameter from 58.44 ± 4.68 µm at a flow rate of 5.0 µL min^−1^ to 77.83 ± 10.29 at a flow rate of 7.5 µL min^−1^, and to 79.99 ± 4.56 µm at a flow rate of 10.0 µL min^−1^ (data represented as mean ± SD) (Figure 2E; Table S2, Supporting Information). A broader particle size distribution was observed at a flow rate of 7.5 µL min^−1^ compared to the other two rates, likely due to the presence of a few outliers with larger or smaller diameters. The ability to produce microgels of varying sizes could benefit cell culture applications, as literature has shown that particle size and void space affect cell spreading and proliferation.^[^ ^47^ ^]^

Lastly, 2.0 w/v% microgels were stored at 37 °C in acidic, physiological, and basic PBS solutions (PBS with a pH of 2, 7.4, and 10, respectively) to assess their stability represented as a function of diameter over time (Figure 2F). Statistical analysis showed no significant difference in diameter for microgels stored in acidic and physiological PBS for a duration of 11 days, compared to their initial size. However, microgels stored in basic conditions for the same period exhibit a slight increase in diameter (*p < 0.05*). This might be attributed to the deprotonation of the hydroxy group of the UPy enol tautomer caused by the alkaline pH, which resulted in a weakened supramolecular network and consequent disassembly of the UPy‐fibers which led to an increase in microgel size by swelling of the microgel network.^[^ ^48^ ^]^ This phenomenon is more often observed in microgels: for example, microgels of poly(acrylic acid) crosslinked with a redox‐sensitive linker showed swelling upon degradation by reduction of the disulfide bridges, while the other way around, hyaluronic acid microgels exhibited a decrease in swelling when the crosslinking density was increased.^[^ ^49^, ^50^ ^]^ For all different pH solutions, the microgels were stable enough to prevent amalgamation into a bulk gel after 11 days.

### 2.3 Mechanical Properties of Micro and Macrogels

**Figure 3:**
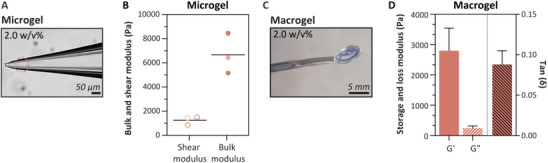
Mechanical properties of microgels and macrogels. A) On a microscopic level we used capillary micromechanics to measure mechanical properties of single microgels. Scale bar represents 50 µm. B) Average shear and bulk modulus of individual microgels (*n* = 3). C) On a macroscopic level we used rheology on 2.0 w/v% macrogels. Scale bar represents 5 mm. D) Average storage and loss modulus, and tan δ of macrogels (*n* = 3).

Here we compared the difference in mechanical properties between 2.0 w/v% microgels and macrogels. On a microscopic level we used capillary micromechanics to measure mechanical properties of single microgels according to the method of Wyss et al., where the response of the microgels to an external applied force was measured (**Figure** **3**A).^[^ ^51^, ^52^ ^]^ Once the microgel was trapped in the tapered section of the glass capillary, it blocked the flow and the entire pressure fell off across the microgel. The pressure was increased and kept constant until the microgel did not move any further in the tapered capillary. At this point, the internal stresses of the microgel were in equilibrium with the externally applied pressure, and images of the deformed microgel in the tapered capillary were taken (Figure S2A, Supporting Information). The process of increasing pressure, waiting for particles to reach an equilibrium and taking images were performed in a semi‐automated fashion in which this sequence was repeated on each microgel for a range of different applied pressures. Characteristic stresses and strains were calculated by the measured geometry of the capillary and the microgel (L_band_ and R_band_) as well as the applied pressure according to Equations (1), (2), (3) (Figure S2B, Supporting Information). In doing so, for each microgel measured, a typical stress‐strain curve was obtained (Figure S2C, Supporting Information; with *n* = 3 microgel). The shear stress was plotted as a function of shear strain, and the slope of the linear fitted line through the data points represents the average shear modulus for each microgel (m_shear_1‐3). The shear modulus, which quantifies the resistance of the material to a shape deformation, showed an average of 1246 ± 344 Pa (Figure 3B). The bulk modulus (also referred to as the osmotic modulus) corresponds to the slope in a plot of the compressive stress as a function of the volumetric strain deformation (Figure S2D, Supporting Information; with *n* = 3 microgels, m_bulk_1‐3). This resulted in an average bulk modulus of 6673 ± 1663 Pa (Figure 3B). On a macroscopic level, we used shear rheology to determine the elastic (G′) and viscous properties (G″) of the 2.0 w/v% macrogel, and a stress relaxation test to quantify the dynamic behavior was performed (Figure 3C; Table S3, Supporting Information). The stress relaxation follows the dissipation of deformation energy via internal rearrangements, which lowers the stress in the structure. The macrogel displayed an averaged relaxed stress of 64.7% during the course of the experiment (Figure S3A, Supporting Information). An average storage and loss modulus of 2798 ± 743 (Pa) and 245 ± 71 (Pa) were measured, respectively (Figure 3D; Figure S3B, Supporting Information).

The mechanical properties of the microgels were measured as shear and bulk moduli, while the macrogels were assessed using the storage modulus. Consequently, direct comparisons between the mechanical properties of microgels and macrogels are not possible. While the methodologies employed for measurements at the microscopic and macroscopic scales differ, we can still conclude that the mechanical properties measured at both scales are in the same order of magnitude of a few kPa. Besides that, literature indicates that the mechanical properties of supramolecular hydrogel systems are affected by the specific volume utilized.^[^ ^53^ ^]^ This could lead to variations in mechanical properties between microgels of ≈100 picoliters and macrogels composed of ≈100 microliters.

**Figure 4:**
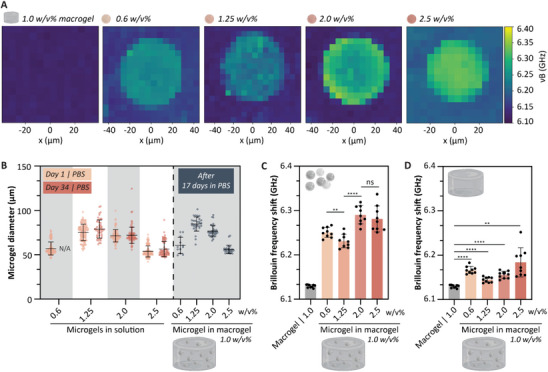
Brillouin microscopy to measure the relative mechanical properties of microgels. A) 3D mapping of Brillouin frequency shifts (ν_B_) of 0.6, 1.25, 2.0, and 2.5 w/v% microgels in a 1.0 w/v% macrogel and an empty 1.0 w/v% macrogel as control. B) Microgel diameter 1 and 34 days after formulation, and after 17 days of formulation embedded in a macrogel. C) Quantified Brillouin frequency shifts of the empty 1.0 w/v% macrogel as a control, and the 0.6, 1.25, 2.0, and 2.5 w/v% microgels in a 1.0 w/v% macrogel. D) Quantified Brillouin frequency shifts of the empty 1.0 w/v% macrogel as a control, and the surrounding matrix of the macrogels after encapsulating 0.6, 1.25, 2.0, and 2.5 w/v% microgels. For all analyses: unpaired t‐test with Welch's correction; **p < 0.01 and ****p < 0.0001; *n* = 3 hydrogels, within each hydrogel 3 microgels measured; values shown as mean ± SD.

Next, Brillouin microscopy was used to characterize the relative mechanical properties of supramolecular microgels. Brillouin microscopy is a type of optical elastography method to characterize local mechanical properties at the cellular and subcellular scale, in 3D, via a non‐destructive, label‐ and contact‐free method.^[^ ^54^, ^55^, ^56^ ^]^ It is based on the principle of Brillouin light scattering, where light from a focused beam is inelastically scattered by the sample's intrinsic thermal fluctuation. As a result the scattered light can undergo a Brillouin frequency shift (BFS), which correlates with the material's elastic properties, with stiffer materials exhibiting a higher frequency shift. Here we compared the mechanical properties of microgels at concentrations of 0.6, 1.25, 2.0, and 2.5 w/v%. Due to the sensitivity of the technique, microgel immobilization was required which was achieved by embedding them in a 1.0 w/v% macrogel, using an empty macrogel as a control. The composite hydrogels were allowed to gelate overnight, after which three microgels from each of three different composites were selected to measure the Brillouin frequency shift (BFS; νB). The BFS values were measured every 6 µm and plotted on a color‐coded Brillouin map, where stiffer materials exhibit a higher frequency shift (**Figure** **4**A). Variations in diameter were observed among the different microgel w/v% concentrations and remained consistent over 34 days solution in PBS, with only the lowest concentration showing dissolution and was therefore not able to be measured (Figure 4B). The microgels were embedded in a 1.0 w/v% macrogel after storage of 17 days in PBS, and their diameter was measured again. An increase in diameter was observed for the 0.6, 1.25, and 2.0 w/v% microgels after encapsulation. The larger diameter of encapsulated microgels compared to those stored in PBS for 34 days suggests an interaction between the microgel and macrogel. Supporting this, the literature indicates that microgels are sensitive to their environment and can interact with their surroundings by adapting their size, shape, and properties.^[^ ^57^ ^]^

Quantified BFS values of the empty 1.0 w/v% macrogel (control) showed a significantly lower shift compared to all microgel conditions, including the lower 0.6 w/v% concentration (Figure 4C). This indicates that microgels exhibit different mechanical properties compared to macrogels. Bulk rheology on macrogels shows that increasing w/v% results in stiffer mechanical properties, as indicated by the G’ value.^[^ ^33^ ^]^ Our results demonstrated that we can formulate microgels with diverse mechanical properties, as we observe a clear difference in mechanical properties between lower and higher w/v% microgels. However, the linear trend observed in bulk hydrogels does not apply at the microgel level. The 0.6 w/v% microgels were stiffer than 1.25 w/v% microgels, and no significant differences in mechanical properties were observed between the 2.0 and 2.5 w/v% microgel. Since the microgel and macrogel are composed of the same molecular building blocks, we expect an interaction between them, with the microgel influencing the properties of the macrogel and vice versa. This could explain that the 1.0 w/v% macrogel enhanced the lower 0.6 w/v% microgel, resulting in a relatively higher BFS than for the 1.25 w/v% microgel. To further investigate this, we analyzed the background representing the macrogel from the same Brillouin maps and plotted the quantified data as the BFS, using the empty 1.0 w/v% macrogel as a control (Figure 4D). The background macrogel for all composites showed a significantly higher BFS compared to the control group, indicating that the presence of microgels at all w/v% concentrations enhanced the mechanical properties of the macrogels. Since we only look a few micron (≈between 10 to 20 µm) near the microgel, we could not conclude if this translates to a bulk effect.

### 2.4 Tuning the Microgel Bioactivity

**Figure 5:**
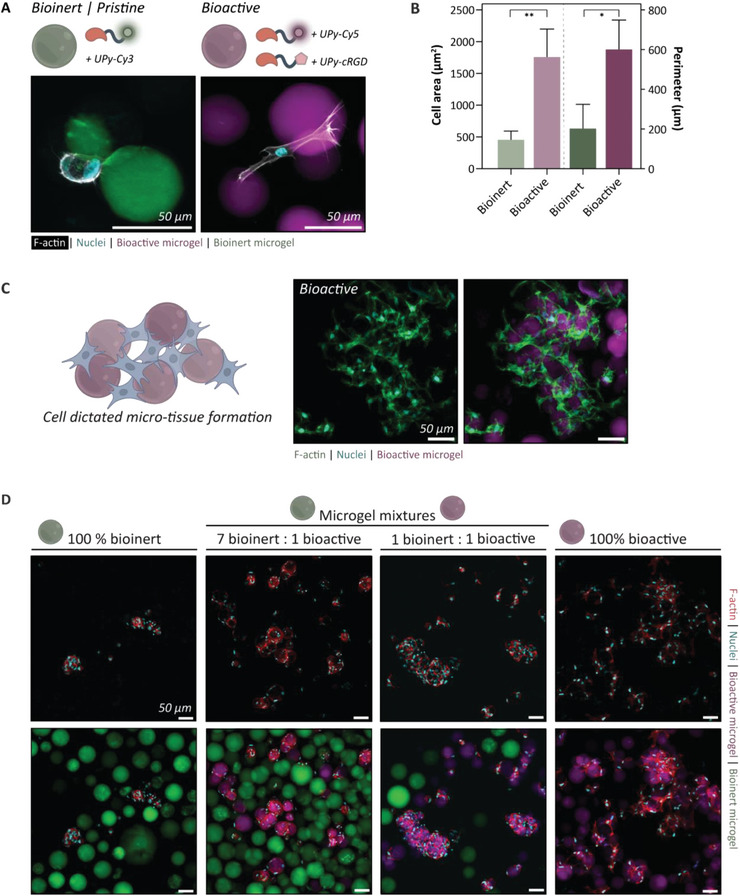
Tuning the microgel bioactivity. A) Schematic representation of 2.0 w/v% microgels functionalized with the integrin‐binding UPy‐cRGD and UPy‐Cy5 for cell adhesion (bioactive), or microgels with solely UPy‐Cy3 (bioinert). Normal human dermal fibroblasts (NHDF) were cultured on either bioactive or bioinert microgels. Cell spreading was observed for NHDF cultured on bioactive microgels, while cells stay small and round when cultured on bioinert microgels. F‐actin in white, nuclei in cyan, and bioactive and bioinert microgels in magenta and green, respectively. B) This was confirmed by a larger cell area and perimeter for cells cultured on bioactive microgels. Statistical analysis showed a significant difference between both perimeters and areas with *p < 0.05 and **p < 0.01 (Kolmogorov‐Smirnov test; *n* = 5; values shown as mean ± SD), respectively. C) NHDF on bioactive microgels self‐assembled into microtissue via cell‐cell and cell‐material interactions, after one day of culture. F‐actin in green, nuclei in cyan and microgel in magenta. D) NHDF cultured on either bioactive or bioinert microgels showed cell spreading and microgel assembly for cells on bioactive microgels. NHDF cultured on a mixture of bioactive and bioinert microgels in the ratio 1:1 or 1:7, respectively, showed solely cell adherence to the bioactive microgels, after one day of culture. For all images: F‐actin in red, nuclei in cyan, and bioinert and bioactive microgels in green and magenta, respectively. All scale bars represent 50 µm.

Normal human dermal fibroblasts (NHDF) were used to explore the possibilities of cell culture on the supramolecular microgels. To this end, bioactive microgels functionalized with the integrin‐binding UPy‐functionalized cyclic arginine‐glycine‐aspartate ligand (UPy‐cRGD; 1.5 mM) for cell adhesion were fabricated with an additional UPy‐functionalized dye UPy‐Cy5. Microgels solely enriched with a UPy‐Cy3 dye were used as bioinert microgels (Table S2, Supporting Information). The UPy‐additives were co‐dissolved in the M‐type hydrogel precursor and mixed on chip with the B‐type precursor to be integrated in the microgel. After 1 day of culture, NHDF on the bioactive microgels demonstrated distinctive spreading behavior in comparison to NHDF cultured on bioinert microgels (3000 microgels and 3000 cells per well; ratio 1:1) (**Figure** **5**A). This observation was supported by quantifying a higher cell area and perimeter for cells adhered to bioactive microgels (Figure 5B). A remarkable finding is that the formation of the self‐assembled microconstruct within a single day of culture on bioactive microgels is exclusively driven by cell‐cell and cell‐material interactions (Figure 5C). This phenomenon is completely cell‐driven and results in the generation of void spaces between the spherical microgels forming a microporous scaffold stimulating cellular growth and tissue formation. This phenomenon can even be tuned by mixing various cell/microgel ratios (Figure S4, Supporting Information). Increasing the ratio cells to microgels results in the formation of a denser self‐assembled scaffold (750 microgels and 3000 cells per well; ratio 1:4) compared to a 1:1 ratio of cells to microgels.

### 2.5 Cellular Selectivity toward Microgel Compositions

The abovementioned results showed the ability of cells to sense the presence of a specific cell adhesive additive and migrate or adhere accordingly. This phenomenon was further investigated by exposing cells not only to individual microgel conditions but also to combinations of bioinert and bioactive microgels within a single culture well. To this end, NHDF were cultured on microgels (3000 microgels and 3000 cells per well; ratio 1:1) for one day that was exclusively bioactive or bioinert, which confirmed similar cellular behavior as described previously (Figure 5D; 100% bioinert and 100% bioactive). While the 100% bioinert condition did reveal the presence of some cells, these were primarily cells adhering to each other through cell‐cell interactions rather than attaching to the material through cell‐material interactions. Then, NHDF were cultured on a mixture of bioactive and bioinert microgels in ratios of 1:1 and 1:7, respectively, while maintaining a constant total number of microgels and cells. The cells exhibited the ability to discern between both microgel conditions, selectively adhering solely to the bioactive microgels, even in the presence of a significantly higher quantity of bioinert microgels (Figure 5D). Additionally, this phenomenon was investigated using fluorescent time‐lapse imaging, wherein bioinert microgels were labeled with a green fluorescent marker, while bioactive microgels were visualized through brightfield microscopy (Video S1 and Figure S5, Supporting Information). Three distinct cell populations were identified based on the captured images: (1) cells adhering directly to a bioactive microgel and spreading (white box), (2) cells initially adhering to a bioinert microgel but capable of reaching a bioactive microgel and initiating spreading (black box), and (3) cells remaining rounded on the bioinert microgel (red box). The cells demonstrated the capacity to distinguish between the two microgel conditions by migrating toward the bioactive microgels when these were present in close proximity.

Over time, a mixture of 2.0 w/v% microgels functionalized with a UPy‐Cy3 or UPy‐Cy5 dye revealed the exchange of UPy‐Cy3 and UPy‐Cy5 from one microgel to another after 7 days of incubation (Figure S6A, Supporting Information). This is caused by the intrinsic dynamic properties of the hydrogel system directed through the non‐covalent interactions between the UPy‐building blocks.^[^ ^29^ ^]^ The dynamic UPy‐Cy3 and UPy‐Cy5 exchange was quantified after day 2 and 7. The total fluorescence intensity of each microgel was measured and the portion corresponding to the UPy‐Cy3 and UPy‐Cy5 dyes was determined and plotted as a normalized fraction of UPy‐Cy3 and UPy‐Cy5 within each microgel (Figure S6B, Supporting Information). After 7 days, more UPy‐dye was exchanged, as indicated by the increased normalized fraction compared to day 2, with UPy‐Cy5 exchange rising from a maximum of 0.1 to 0.14, and UPy‐Cy3 exchange increasing from a maximum of 0.19 to 0.31. Likely, it is believed that dynamic exchange of UPy‐monomers also extends to the cell adhesive UPy‐cRGD, allowing it to migrate from a bioactive to bioinert microgel. This would enhance the cell‐adhesive properties of the bioinert microgel, thereby making it more attractive for cell adhesion. However, this effect appears to have minimum impact, as the cells still predominantly adhere to the bioactive microgels after 7 days of culture (Figure S6C, Supporting Information).

### 2.6 Microgels Embedded in a Supramolecular Macrogel

#### 2.6.1 Microgels Embedded in a Robust Supramolecular Macrogel

**Figure 6:**
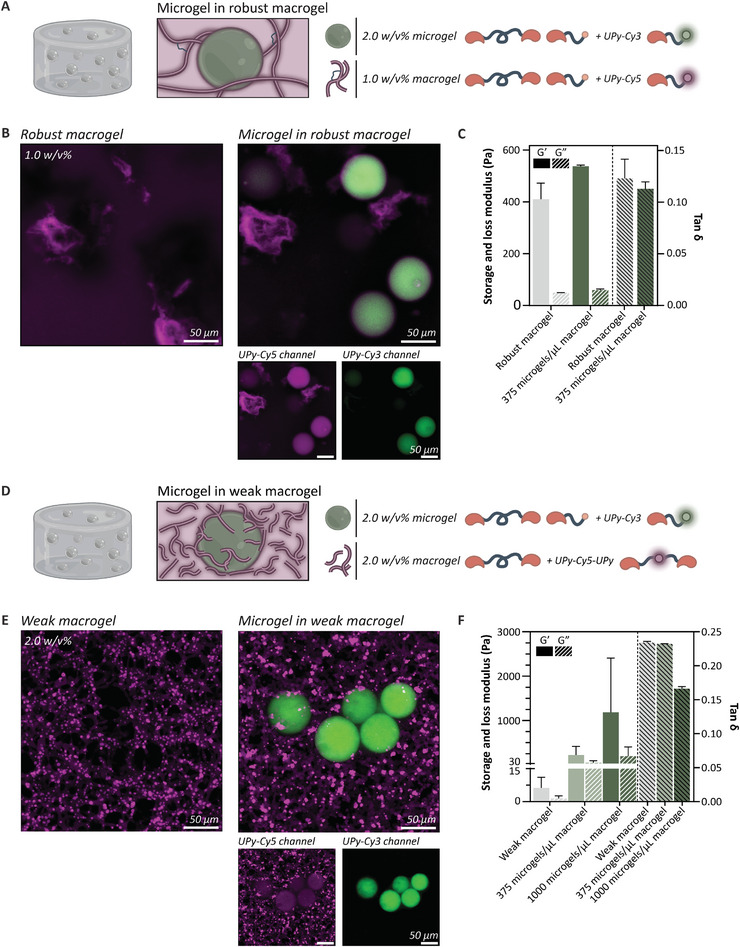
Microgels embedded in a supramolecular macrogel A) Schematic overview of a multicompartment hydrogel system established by the integration of 2.0 w/v% microgels (modified with UPy‐Cy3) within a 1.0 w/v% macrogel composed of B‐ and M‐type molecules (modified with UPy‐Cy5). B) Confocal images revealed the distinct microgel compartment (green) in the macrogel (magenta). Scale bars represent 50 µm. C) Microgel incorporation enhanced the storage modulus of the macrogel (G’) while unaffecting the viscoelastic properties (tan δ). D) A multicompartment hydrogel system was established by the integration of 2.0 w/v% microgels (modified with UPy‐Cy3; green) within a 2.0 w/v% supramolecular macrogel composed of solely B‐type molecules (modified with UPy‐Cy5‐UPy; magenta). E) The B‐type molecule itself formed a fibrous network and the addition of microgels showed distinct compartmentalization of the microgel in the macrogel. Scale bars represent 50 µm. F) Microgel incorporation significantly enhanced the storage modulus of the macrogel (G’).

Next, a multicompartment hydrogel system was established by the integration of microgels within a macrogel composed of B‐ and M‐type molecules, which is a robust hydrogel with slow stress relaxation.^[^ ^29^ ^]^ Here, the same supramolecular building blocks were used for both the micro and macrogel, which allows to modularly build composite hydrogels with independent control of micro and macrogel compartments, offering spatiotemporal control over the cellular microenvironment. For this end, 2.0 w/v% microgels were formulated and as a proof of concept functionalized with the function of a fluorescent UPy‐Cy3 additive. They were mixed at a concentration of 375 microgels µL^−1^ hydrogel into a 1.0 w/v% macrogel enriched with an UPy‐Cy5 dye or UPy‐Cy5‐UPy (Table S3, **Figures** **6**A and S7A,B, Supporting Information, respectively). Theoretically, this will increase the macrogel concentration to 1.1 w/v%. Confocal images reveal the distinct microgel compartment (green) in the macrogel (magenta) (Figure 6B). The M‐type building block of the macrogel seems to condensate on the microgel, indicative by the overlap of UPy‐Cy5 on the UPy‐Cy3 signal. Conversely, the transfer of M‐type molecules from the microgel to the macrogel was less prominent, as no UPy‐Cy3 was overlapping in the network of UPy‐Cy5 signal. Over a span of 7 days, the microgels did not fuse with the macrogel, substantiated by the absence of changes in microgel diameter (Figure S8A–E, Supporting Information). Additionally, the exchange of M‐type molecules from the microgel into the macrogel network remained limited, indicating that we have created stable micro domains in a macrogel.

Rheological measurements revealed that incorporation of microgels within the bulk hydrogel led to a small augmentation in overall bulk stiffness, as evidenced by an elevated storage modulus (G'), while the viscoelastic properties of the hydrogel remained unaffected (Figure 6C). This phenomenon is attributed to the hypothesis that the introduction of preformed microgels into the hydrogel precursor solution imparts supplementary crosslinking, which leads to mechanical reinforcement of the macrogel. In conventional pristine hydrogels, the crosslinking of M‐type fibers in the precursor solution is facilitated by B‐type molecules. However, in the context of microgel incorporation, it is proposed that the existing microgels serve as supplementary crosslinkers during the formation of the macrogel network. Consequently, the macrogel is directly established and constructed around the microgel interphase and the dynamic and modular behavior intrinsic to supramolecular building blocks enables the stable integration of microgels within the macrogel. This serves as a modular strategy for generating compartmentalized architectures within hydrogel matrices, and enables the creation of intricate and multiphasic architectures which holds considerable promise for diverse in vitro applications.

#### 2.6.2 Microgels Embedded in a Weak Supramolecular Macrogel

Next, a multicompartment hydrogel system was established by the integration of microgels within a supramolecular macrogel composed of solely B‐type molecules, which is a dynamic and weak hydrogel with high stress relaxation.^[^ ^29^ ^]^ Complexity can be added into this macrogel by the introduction of microgels. For this end, 2.0 w/v% microgels were formulated and as a proof of concept functionalized with the function of a fluorescent UPy‐Cy3 additive. They were mixed into a 2.0 w/v% hydrogel enriched with an UPy‐Cy5‐UPy dye (Figure 6D). The B‐type molecule itself displays high dynamic behavior attributed to the fast exchange on a molecular level and forms a fibrous network at this concentration (Figure 6E). The integration of microgels in the macrogel resulted in a decrease in pore size of the macrogel. This might be due to a reduced dynamic behavior of the B‐type network and the formation of a fibrous network around the microgels that serve as additional crosslink sites, as the UPy‐Cy5‐UPy signal was found to condensate at the microgel interphase. This is attributed to the dynamic supramolecular interactions between the microgel and the weak dynamic B‐type macrogel.

For the rheological measurements, a 2.0 w/v% B‐type macrogel was used that on its own is a very soft hydrogel of only a few Pascal (6.3 ± 4.8 Pa) (Figure 6F). When mixing 375 microgels µL^−1^ hydrogel into the macrogel, an almost 35‐fold increase in bulk stiffness (223 ± 195 Pa) was observed, while the viscoelastic properties of the hydrogel remained unaffected. This effect was enhanced by mixing 1000 microgels µL^−1^ hydrogel into the macrogel, where a bulk stiffness of 1185 ± 1223 Pa was obtained. However, the tan δ dropped, indicating a change in viscoelastic behavior. Furthermore, the error bars for the composite hydrogel are significantly large. Supramolecular hydrogels inherently exhibit some heterogeneity, as evidenced by confocal images showing regions with varying concentrations of UPy‐dye. Additionally, mixing microgels with macrogel precursor solutions may introduce further heterogeneity, as the microgels are randomly distributed throughout the composite. Furthermore, when using microgels at a higher concentration this effect is enhanced.

In general, the dynamic and modular behavior intrinsic to supramolecular building blocks used in the micro and macrogel enables the reinforcement of a weak macrogel by providing crosslinking points to strengthen the macrogel. Furthermore, it allows the functionalization of weak and dynamic hydrogels with distinct microgel compartments.

## 3 Conclusion

We succeeded in stable supramolecular microgel formulation exclusively using supramolecular monomers. At the nanoscale the modular characteristics of the molecular building blocks facilitate accurate adjustment of microgel properties. This approach enables the formulation of microgels with adjustable B‐ to M‐type monomer ratios that dictates their dynamics, as well as varying monomer concentrations that define their mechanical properties. Microgels functionalized with a bioactive supramolecular cell‐adhesive RGD‐peptide support cell culture and show selectivity of cells toward bioactive microgels over non‐functionalized versions when both conditions are present in the same culture well. This observed cellular distinctiveness holds promise for future cost‐effective screening of multiple material compositions at a microscale level. Supramolecular microgels can be applied as defined modular micro‐building blocks in supramolecular macrogels to achieve precise individual spatial control over both the micro‐ and macro‐environment, and the ability to reinforce and enhance mechanical properties of macrogels. With this approach, we successfully established modular control across length scales, from the molecular to the nano, micro and macroscale.

## 4 Experimental Section

### Materials

All reagents, chemicals, materials and solvents were obtained from commercial sources and were used as received. Phosphate‐buffered saline (PBS), polydimethylsiloxane (PDMS), PDMS curing agent, mineral oil, formalin 37%, phalloidin‐488, 4′,6‐diamidino‐2‐phenylindole (DAPI), Triton X‐100 were purchased from Merck. SU‐8 3000 photoresist was purchased from Micro Resist Technology, 1H,1H,2H,2H‐perfluorooctyltriethoxysilane and fluorinated HFE‐7500 from Fluorchem, Pico‐Surf from Sphere Fluidics, 1H,1H,2H,2H‐perfluoro‐1‐octanol (PFO) from Sigma‐Aldrich, normal human dermal fibroblasts (NHDF) from Lonza, fetal bovine serum (FBS) from Greiner Bio‐one, penicillin/streptomycin from Invitrogen, and Ibidi‐slide (Ibidi 15 µ‐Slide Angiogenesis glass bottom) from Ibidi. DMEM medium, trypsin/EDTA, 96 non‐adhesive u‐bottom well plate (Scientific, Nunclon Sphera‐Treated, U‐Shaped‐Bottom plate), and 8 well chamber slide (Nunc Lab‐Tek Chamber) were purchased from Thermo Fisher Scientific. Images were acquired using Leica TCS SP8 X inverted confocal microscope (Leica Microsystems) using HC PL APO CS2 objectives (20x/0.75, 40x/0.95) and processed in ImageJ. Statistical analysis were performed in GraphPad Prism 8.0.2 software.

### Microfluidic Device Production

Microfluidic devices were manufactured from PDMS molds using soft lithography. Photomasks for soft photolithography were ordered from CAD/Art Services, Inc. (Bandon, Oregon) as previously used and described in Sinha et al.^[^ ^40^ ^]^ PDMS molds were produced by spin‐coating wafers with SU‐8 3000 photoresist according to manufacturer's protocol to obtain 30 µm of channel height. Microfluidic devices were fabricated by mixing PDMS base and curing agent at a ratio of 10:1 w/w%. After air bubble removal with vacuum, the mixture was poured onto a master silicon wafer containing the device layout (Figure S1A, Supporting Information) and the mixture was cured at 65 °C for 3 h. After curing, the PDMS was carefully removed from the wafer and 1 mm holes were punched for the inlets and outlet. The PDMS was bonded channels‐down to glass slides to yield closed microchannels via OH‐terminated by exposure to plasma (Emitech K1050X), whereafter the devices were incubated at 65 °C for 1 h to increase binding. Finally, channels were treated with 5 v/v% 1H,1H,2H,2H‐ perfluorooctyltriethoxysilane in fluorinated HFE‐7500, incubated at 65 °C for 1 h, flushed with HFE‐7500, and incubated overnight at 65 °C.

### Microgel Formation

Microgels composed of supramolecular building blocks were generated using a tip‐loading approach, as previously described in Sinha et al.^[^ ^40^ ^]^ Pre‐solutions of bi‐ and monofunctional UPy‐molecules were prepared separately (Tables S1,S2, Supporting Information). Monofunctional UPy‐compound was dissolved at 70 °C for 1 h under stirring conditions in an alkaline solution (i.e., 80 or 160 mM NaOH in PBS if the pre‐solution was < 5 w/v% or ≥ 5 w/v%, respectively). The resulting solution was neutralized by the addition of acid (i.e., 1 M HCl or 2 M HCl, respectively to the amount of base) and then diluted with PBS. For fluorescent microgels, UPy‐Cy3 or UPy‐Cy5 was added to this solution from a 1.23 mM DMSO stock. The bifunctional molecule was dissolved at 70 °C in PBS for 1 h. Subsequently, the pre‐solutions were separately drawn into a 200 µL pipette tip and loaded into the microfluidic chip according to Figure S1A (Supporting Information). Syringes and tubing filled with mineral oil were used as hydraulic system to dispense the liquids from the syringes driven by computer‐controlled pumps (neMESYS microfluidic pump, Cetoni). HFE‐7500 with 2.5 v/v% Pico‐Surf was flushed into the oil inlet to form microgels. Microgel production commenced with an oil flow rate of 10 µL min^−1^ to prefill the channels of the microfluidic device. Then, the flow of the hydrogel pre‐solutions was started simultaneously at 5 µL min^−1^ (larger microgels could be formulated by increasing the pre‐solution flow to 10 µL min^−1^). Once the hydrogel pre‐solutions started to mix at the droplet formation point, the oil flow was increased to 30 µL min^−1^. The droplets were collected in an Eppendorf tube from the outlet, and PBS was poured on top of the emulsion to prevent evaporation of the HFE oil. The collected droplets were then incubated at 37 °C for 3 h to facilitate gelation of the microgel. Thereafter, the microgels were isolated by demulsification with 20 v/v% PFO in HFE‐7500 oil. Subsequently, the microgels were collected, resuspended in fresh medium or PBS, and counted using a hemocytometer.

### Capillary Micromechanics

A flow of 2.0 w/v% microgels in PBS was generated by applying a pressure difference between the inlet and outlet of a tapered capillary according to a previously described method.^[^ ^51^, ^52^ ^]^ The tip diameter and length of the taper were controlled by the parameters of the pipette puller (Sutter instruments P‐97). A modular pressure‐based flow controller was used (Fluigent Flow EZ LU‐FEZ‐2000) to apply pressure difference between the inlet and outlet of the tapered capillary. Once a single microgel was trapped in the tapering section of the capillary, it blocked the flow and the entire pressure difference fell across the microgel. When the pressure was increased, the microgel moved toward the tip of the capillary. For the stress‐strain measurements, the pressure was increased and the deformation of the particle was imaged after the microgel had reached equilibrium, using Nikon DS‐Fi3 camera mounted on a Nikon Ts2R inverted microscope. Thereafter, the stresses were computed using following equations (Equations (1), (2), (3), (4), (5), (6), (7)) via a previous described method and the stress was plotted as a function of strain.^[^ ^51^, ^52^ ^]^

(1) $$\;F_{||wall}=2\pi R_{band}L_{band}P_{wall}sin\left(\alpha \right)\;\left[N\right]$$

(2) $$\;F_{\left|\right|}=p\pi R_{band}^{2}\;\left[N\right]$$

(3) $$\;P_{wall}=\frac{R_{band}}{2L_{band}\sin\left(\alpha \right)}\;p\;\left[\mathrm{Pa}\right]$$

(4) $$\;\sigma_{r}=K\left(2\varepsilon_{r}+\varepsilon_{z}\right)+\frac{2}{3}G\left(2\varepsilon_{r}-\varepsilon_{z}\right)\;\left[\mathrm{Pa}\right]$$

(5) $$\;\sigma_{r}=K\left(2\varepsilon_{r}+\varepsilon_{z}\right)-\frac{4}{3}G\left(2\varepsilon_{r}-\varepsilon_{z}\right)\;\left[\mathrm{Pa}\right]$$

(6) $$K=\frac{\frac{1}{3}\left(2p_{wall}+p\right)}{2\varepsilon_{r}+\varepsilon_{z}}\;\;\left[\mathrm{Pa}\right]$$

(7) $$G=\frac{\frac{1}{2}\left(p_{wall}-p\right)}{\varepsilon_{r}-\varepsilon_{z}}\;\;\left[\mathrm{Pa}\right]$$

### Microgel Stability

Microgels (2.0 w/v%) were fabricated using the previously described method. Then, microgels were isolated from the oil phase and resuspended in PBS solutions with pH values of 2, 7.4, and 10. The microgel diameter was determined from brightfield images at day 1, 2, 8, and 11 for all conditions in ImageJ.

### Brillouin Microscopy

A 1.0 w/v% UPy‐macrogel was prepared by dissolving the B‐type molecule at 0.2 w/v% in PBS and the M‐type molecule was dissolved at 3.6 w/v% in 80 mM NaOH in PBS, both at 70 °C for 1 h. The M‐type solution was neutralized with 1 M HCl, diluted to 1.8 w/v% with PBS. Microgels were prepared as described previously and were shipped to the Advanced Research Centre, University Glasgow. Due to shipment, microgels had to be stored for 17 days in PBS after they were encapsulated in the macrogel by mixing in 375 microgels per total µL hydrogel in the B‐type solution, which was mixed 1:1 with the M‐type solution. A pristine macrogel without microgels was used as a control 85 µL of both pristine and microgel‐in‐macrogel mixtures was pipetted in a parafilm dimple (⌀ 8 mm) and were gelated overnight at 37 °C before measuring. Brillouin maps were acquired using a confocal Brillouin microscope employing a VIPA‐based spectrometer (hyperfine spectrometer, Light Machinery, Canada) with a tunable filter to suppress the main laser peak (pump killer). Samples were placed in NuncTM glass‐bottom petri dishes (35 mm) and were illuminated with a single mode laser (Cobolt Flamenco, Hubner Photonics) at a wavelength of 660 nm. The laser power at the sample position was ≈50 mW using the OD0.3 filter. All images were obtained with a 20×0.45 NA objective at room temperature (*n* = 3 hydrogels, within each hydrogel 3 microgels measured).

### Cell Culture

Normal human dermal fibroblasts (NHDF; Lonza) were cultured under standard culturing conditions at 37 °C and 5% CO_2_ in Dulbecco's Modified Eagle Medium (DMEM) with high glucose, pyruvate and glutaMAX, supplemented with 10 v/v% FBS, and 1 v/v% penicillin/streptomycin. Cells were harvest every 3 days using trypsin/EDTA and cells were used for experiments up to passage 16.

### Cell Culture on Microgels

Microgel with or without UPy‐cRGD (Table S2, Supporting Information) were formulated as described previously to generate bioactive or bioinert microgels, respectively. Per condition, 3000 microgels were pipetted in a 96 non‐adhesive u‐bottom well plate and UV‐sterilized for 20 min. NHDF were harvested and 3000 cells per well were pipetted on top of the microgels and cultured overnight at physiological conditions. Then, microgels were washed 3 times with PBS and fixated at room temperature using 3.7% paraformaldehyde (formalin 37% in PBS) for 20 min. Samples were washed with PBS and permeabilized with 0.5% Triton X‐100 in PBS for 15 min. Then, the cells were stained with phalloidin (1:300) for 40 min and with DAPI (1:250) for 10 min. The cells were washed 3 times with PBS and transferred to an 8 well chamber for imaging purposes. Images were acquired using a confocal microscope. Additionally, various microgel concentrations in combination with cell numbers were prepared and cultured as described above: 1500 cells per 1500 microgels, 3000 cells per 750 microgels, 3000 cells per 1500 microgels, 3000 cells per 3000 microgels, and 3000 cells without microgels. The cells were cultured for 7 days whereafter brightfield images were taken.

### Cell Culture on Mixed Microgels

Microgels (Table S2, Supporting Information) were prepared separately as described earlier. After counting the microgels, suspensions in complete medium were prepared of solely bioinert and bioactive microgels, as well as mixtures of both in different ratios (bioactive:bioinert). In total 3000 microgels per condition were pipetted in a non‐adhesive 96 well plate. Thereafter, NHDFs were seeded and cultured as described above. Time‐lapse imaging of NHDFs was performed in an incubator at 37 °C using a CytoSMART Lux3 FL (CytoSMART Technologies B.V., Eindhoven, The Netherlands). Images were acquired at 20x digital zoom every 5 min for 8 h in brightfield and fluorescent mode with excitation of 452/45 nm and emission 512/23 nm. Thereafter, the cells were fixated, stained, imaged, and processed as described above. Additionally, various microgel concentrations in combination with cell numbers were prepared and cultured as described above: 1500 cells per 1500 microgels, 3000 cells per 750 microgels, 3000 cells per 1500 microgels, 3000 cells per 3000 microgels, and 3000 cells without microgels. The cells were cultured for 7 days whereafter brightfield images were taken.

### Dynamic Exchange between Microgels

B1M84 microgels with UPy‐Cy3 or UPy‐Cy5 additive (Table S1, Supporting Information) were prepared separately as described earlier. Equal amount of UPy‐Cy3 or UPy‐Cy5 functionalized microgels were mixed 1:1. Non‐mixed microgels were used as a control. After 2 and 7 days, confocal images were taken. A custom‐made pipeline in CellProfiler^TM^ was used to measure the total fluorescence intensity of each microgel. The portion corresponding to the UPy‐Cy3 and UPy‐Cy5 dye was determined as well, and plotted as a normalized fraction of UPy‐Cy3 and UPy‐Cy5 within each microgel.

### Microgels in a Dynamic (B‐type) Macrogel

The B‐type molecule was dissolved at 2.0 w/v% for 1 h at 70 °C in PBS, for confocal or rheological measurements. Thereafter, 0.1 mM bivalent UPy‐Cy5‐UPy dye was added for the confocal samples (Table S3, Supporting Information). Microgels were fabricated as described previously (Table S2, Supporting Information). After isolation, the microgels were resuspended (375 or 1000 microgels per total µL hydrogel) in the bifunctional solution. For confocal measurements, 15 µL hydrogel with and without microgels was pipetted in an Ibidi‐slide and incubated overnight at 37 °C. Next, the microgels in the bifunctional network were imaged using a confocal microscope. For rheological measurements, 100 µL hydrogel with and without microgels was pipetted in a parafilm dimple (⌀ 8 mm) and gelated overnight at 37 °C in a humidified chamber (*n* = 3).

### Microgel in a Robust (B+M‐type) Macrogel

A 1.0 w/v% UPy‐macrogel was prepared by dissolving the B‐type molecule at 0.4 w/v% in PBS and the M‐type molecule was dissolved at 3.6 w/v% in 80 mM NaOH in PBS, both at 70 °C for 1 h. The M‐type solution was neutralized with 1 M HCl, diluted to 1.8 w/v% with PBS, and UPy‐Cy5 from DMSO was added to reach a final concentration of 0.1 mM. After dissolving, 0.1 mM UPy‐Cy5 or UPy‐Cy5‐UPy dye was added to the B‐type solution (Table S3, Supporting Information). Microgels with UPy‐Cy3 (Table S2, Supporting Information) were prepared as described above. The pristine macrogel was formulated by mixing the B‐ and M‐type molecule in a 1:1 ratio. Microgel‐in‐macrogel samples were formulated by mixing in 375 microgels per total µL hydrogel in the B‐type solution, which was mixed 1:1 with the M‐type solution. 85 µL of both pristine and microgel‐in‐macrogel mixtures was pipetted in a parafilm dimple (⌀ 8 mm) for rheological measurements (*n* = 3) and 15 µL was pipetted in a Ibidi‐slide for confocal microscopy. Hydrogels were gelated overnight at 37 °C before measuring. Next, the samples were imaged using a confocal microscope. For rheological measurements, an additional pristine macrogel of 2.0 w/v% was formulated as described above and dissolved at the initial concentrations of 0.8 w/v% and 7.2 w/v% for the B‐ and M‐type molecules, respectively.

### Mechanical Properties on Macrogels and Microgel‐in‐Macrogels

Rheological measurements on microgel‐in‐macrogel and pristine samples were performed on a TA Instruments Dynamic Hybrid Rheometer 3 equipped with an 8 mm flat steel plate‐plate geometry with a gap of 1000 µm. Low viscosity silicon oil (47 V 100, RHODORSIL) was used around the hydrogel to minimize sample drying. Samples were loaded at 37 °C after which the complex modulus G* (γ = 0.01, ω = 1 rad s^−1^) was measured for 10 min to ensure that samples were at a stable plateau modulus and were not altered or damaged during loading. Subsequent frequency sweep measurements were performed at ω = 0.01 rad s^−1^ to 100 rad s^−1^, at a strain of γ = 0.01. Stress‐relaxation was measured by applying a strain of γ = 0.075 with a strain rise time of 0.09 s and monitoring the stress for 1000 s. The data were normalized using the highest stress generated during the experiments (always after 0.05 s, disregarding data that was shorter than the strain rise time).

### Statistical Analysis

Statistical analyses were carried out using GraphPad Prism 8.0.2 software, with all data presented as mean ± standard deviation (SD). For all experiments, normality was assessed using the Shapiro‐Wilk test. Data related to microgel size based on flow speed and microgel stability were obtained from brightfield images using ImageJ. Since these conditions did not follow a normal distribution, the Kolmogorov‐Smirnov test was applied to identify statistically significant differences between groups. Data concerning the Brillouin microscopy was normally distributed and the unpaired t‐test with Welch's correction was performed to identify statistically significant differences between microgel conditions. For the cell experiments, all image analysis were analyzed from maximum‐intensity z‐projections of confocal image stacks. CellProfilerTM was used to design a pipeline to determine the cell area and perimeter from the phalloidin staining as an indicator for cell spreading on bioinert and bioactive microgels. The data was checked for normal distribution followed by a Kolmogorov‐Smirnov test to compare columns. In all cases, differences were considered as statistically significant when *P* < 0.05.

## Conflict of Interest

Patricia Dankers is the co‐founder and shareholder of spin‐off company VivArt‐X that focusses on women's health using supramolecular materials; especially targeting breast regeneration.

## Supporting information



Supporting Information

Supplemental Video 1

## Data Availability

The data that support the findings of this study are available from the corresponding author upon reasonable request.
