# Supplementary material for: Using a Supramolecular Monomer Formulation Approach to Engineer Modular, Dynamic Microgels, and Composite Macrogels
Source: Adv Mater. 2024 Oct 27;36(50):2405868. doi: 10.1002/adma.202405868 (PMC11636168; doi:10.1002/adma.202405868)
Supplement: Supplementary file 1 — Supporting Information [file ADMA-36-2405868-s002.docx]

Supporting Information

**Using a Supramolecular Monomer Formulation Approach to Engineer Modular, Dynamic Microgels and Composite Macrogels**

*Maritza M. Rovers, Theodora Rogkoti, Bram K. Bakker, Kalpit J. Bakal, Marcel H.P. van Genderen, Manuel Salmeron-Sanchez, Patricia Y.W. Dankers**


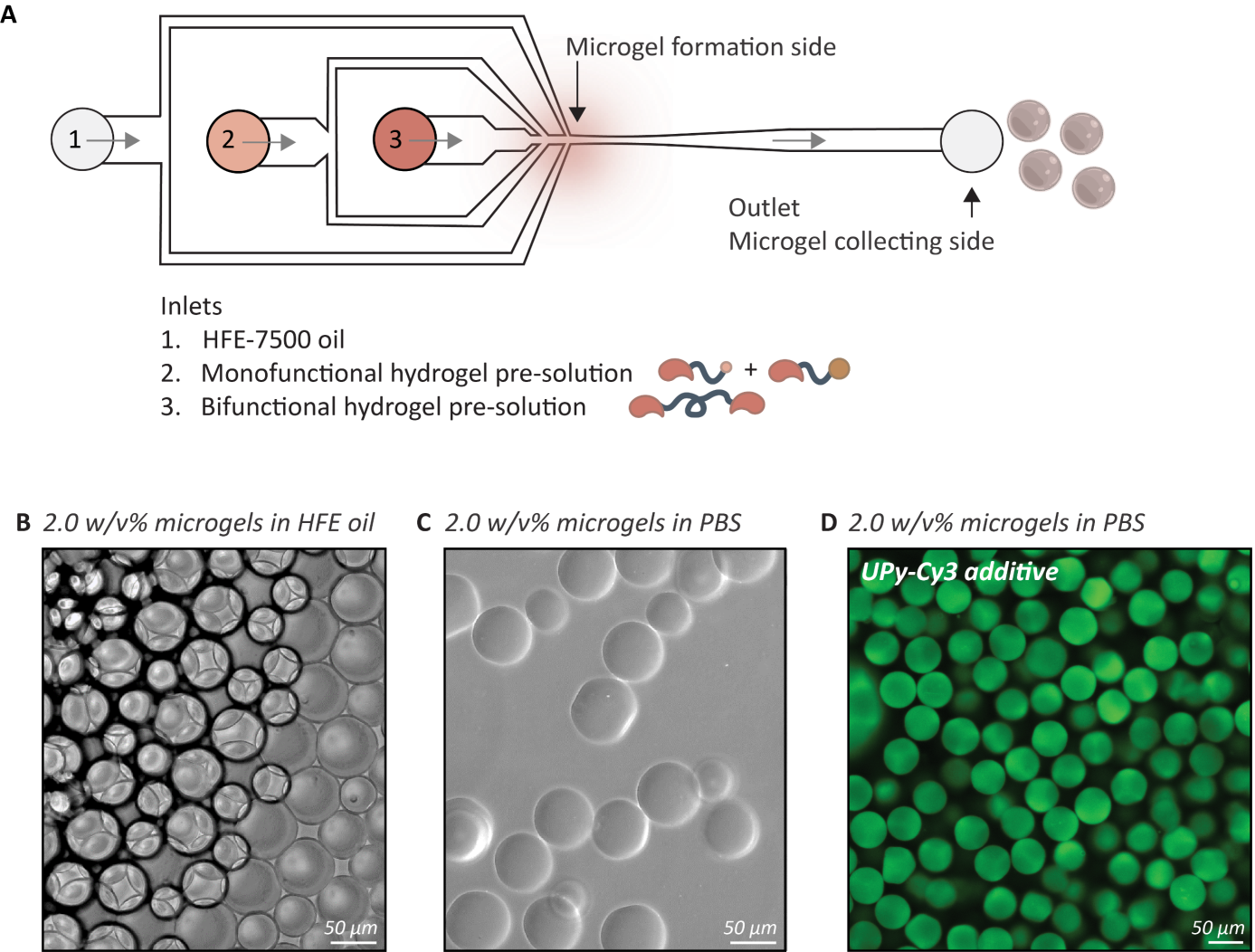


**Figure S1: Microgel production. A)** Schematic representation of the microfluidic chip lay-out to formulate microgels. **B)** 2.0 w/v% microgels in HFE-7500 oil and **C)** the same microgels isolated from the oil in PBS after demulsification. **D)** 2.0 w/v% microgels with UPy-Cy3 in PBS. Scale bars represent 50 μm.

**Table S1:** Table showing the different compositions of 2.0 w/v% microgels formulated with various molar ratios between M- and B-type molecules. ^a^ UPy-Cy5 was added from a 1.23 mM stock in DMSO.

| *Sample ID* | *B-type: UPy_2_-PEG_10K_* | | | *M-type: UPy-Gly + additive* | | | | *Total in microgel* |
| --- | --- | --- | --- | --- | --- | --- | --- | --- |
|  | mM - w/v% - mol% | | | mM - w/v% - mol% | | | |  |
| B1M370 | 0.05 | 0.05 | 0.3 | UPy-Gly  UPy-Cy5 ^a^ | 16.65 | 1.95 | 99.1 | 16.8 mM \| 2.02 w/v% |
|  |  |  |  |  | 0.1 | 0.02 | 0.6 |  |
| B1M84 | 0.18 | 0.20 | 1.18 | UPy-Gly | 15.09 | 1.80 | 98.17 | 15.4 mM \| 2.02 w/v% |
|  |  |  |  | UPy-Cy3/Cy5 ^a^ | 0.1 | 0.02 | 0.65 |  |
| B1M9 | 0.91 | 1.0 | 9.62 | UPy-Gly | 8.45 | 1.0 | 89.32 | 9.46 mM \| 2.02 w/v% |
|  |  |  |  | UPy-Cy5 ^a^ | 0.1 | 0.02 | 1.06 |  |

**Table S2:** Table showing the different microgel compositions used throughout this research, where the ratio between B- and M-type molecules is always B1:M84, respectively. ^a^ UPy-Cy3 and UPy-Cy5 were added from a 1.23 mM stock in DMSO.

| *Sample ID* | *B-type:*  *UPy_2_-PEG_10K_* | | | *M-type: UPy-Gly + additive* | | | | | | *Total in microgel* |
| --- | --- | --- | --- | --- | --- | --- | --- | --- | --- | --- |
|  | mM - w/v% - mol% | | | mM - w/v% - mol% | | | | | |  |
| 0.6 w/v% | 0.05 | 0.06 | 1.18 | 4.55 | 0.54 | | | 98.82 | | 4.6 mM \| 0.6 w/v% |
| 1.0 w/v% | 0.09 | 0.10 | 1.18 | 7.58 | 0.91 | | | 98.82 | | 7.67 mM \| 1.01 w/v% |
| 1.25 w/v% | 0.11 | 0.13 | 1.18 | 9.48 | 1.13 | | | 98.82 | | 9.59 mM \| 1.26 w/v% |
| 2.0 w/v% | 0.18 | 0.20 | 1.18 | 15.19 | 1.82 | | | 98.82 | | 15.37 mM \| 2.02 w/v% |
| 2.5 w/v% | 0.23 | 0.25 | 1.18 | 19.01 | 2.25 | | | 98.82 | | 19.24 mM \| 2.5 w/v% |
| 2.0 w/v%  - bioactive | 0.18 | 0.20 | 1.18 | UPy-Gly | | 13.59 | 1.62 | | 88.42 | 15.37 mM \| 2.10 w/v% |
|  |  |  |  | UPy-Cy5 ^a^ | | 0.1 | 0.02 | | 0.65 |  |
|  |  |  |  | UPy-cRGD | | 1.5 | 0.26 | | 9.75 |  |
| 2.0 w/v%  - UPy-Cy3/Cy5  - bioinert | 0.18 | 0.20 | 1.18 | UPy-Gly | | 15.09 | 1.80 | | 98.17 | 15.37 mM \| 2.02 w/v% |
|  |  |  |  | UPy-Cy3/Cy5 ^a^ | | 0.1 | 0.02 | | 0.65 |  |


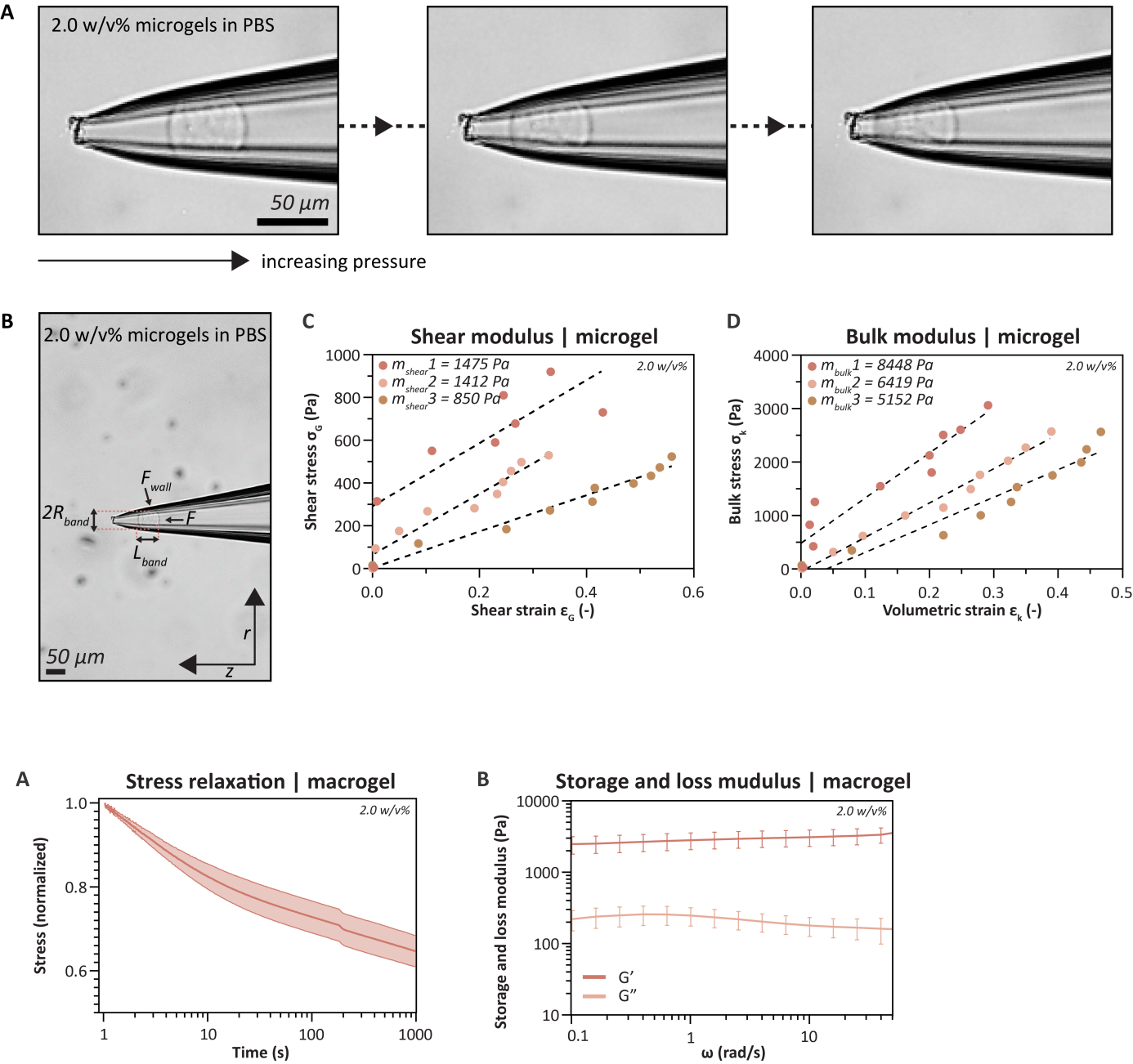


**Figure S2: Mechanical properties of microgels. A)** Microgel deformation in capillary when applied pressure increases. **B)** 2.0 w/v% microgels trapped in the microcapillary. Forces experienced by the particle, F and 𝐹_wall_ , and the effective 𝐿_band_ and 𝑅_band_ , as mentioned in Wyss et al., are indicated.^[54]^ **C)** A typical shear stress graph is plotted as a function of shear strain where the slope through the data points represents the average shear modulus for each microgel (n = 3; m_shear_1-3). **D)** The bulk stress is measured as a function of volumetric strain, where the slope for each microgel represents the average bulk modulus for the microgel (n = 3 ; m_bulk_1-3).

**Table S3:** Table showing the different macrogel compositions used in this study. Encapsulated microgels were always of 2.0 w/v% microgels with a fixed ratio of B- to M-type molecules (B1M84), either with or without UPy-Cy3 dye. ^a^ UPy-Cy5-UPy or UPy-Cy5 was added to the macrogel from a 1.23 mM stock in DMSO.

| *Sample ID* | *Microgels* | | *B-type: UPy_2_-PEG_10K_*  *+ additive* | | | | *M-type:*  *UPy-Gly + additive* | | | | | | | | *Total in macrogel* |
| --- | --- | --- | --- | --- | --- | --- | --- | --- | --- | --- | --- | --- | --- | --- | --- |
|  |  |  | mM - w/v% - mol% | | | | mM - w/v% - mol% | | | | | | | |  |
| 1.0 w/v% robust macrogel  - with/without microgels | - | | 0.09 | 0.10 | | 1.16 | UPy-Gly | | | 7.58 | | 0.91 | | 97.55 | 7.77 mM \| 1.03 w/v% |
|  | 375 /μL gel | |  |  |  |  | UPy-Cy5^a^ | | | 0.1 | | 0.02 | | 1.29 |  |
| 2.0 w/v% robust macrogel | - | | 0.18 | 0.20 | | 1.18 | 15.2 | | 1.82 | | | | 98.82 | | 15.38 mM \| 2.02 w/v% |
|  |  | |  | | | | | | | | | | | |  |
| *Sample ID* | | *Microgels* | | | *B-type: UPy_2_-PEG_10K_ + additive* | | | | | | | | | | *Total in macrogel* |
|  |  |  |  |  | mM - w/v% - mol% | | | | | | | | | |  |
| 2.0 weak macrogel  - with/without microgel | | - | | | UPy_2_-PEG_10K_  UPy-Cy5-UPy^a^ | | | 1.84  0.05 | | | 2.0  0.02 | | | 97.35  2.65 | 1.89 mM \| 2.02 w/v% |
|  |  | 375 /μL gel | | |  |  |  |  |  |  |  |  |  |  |  |
|  |  | 1000 /μL gel | | |  |  |  |  |  |  |  |  |  |  |  |


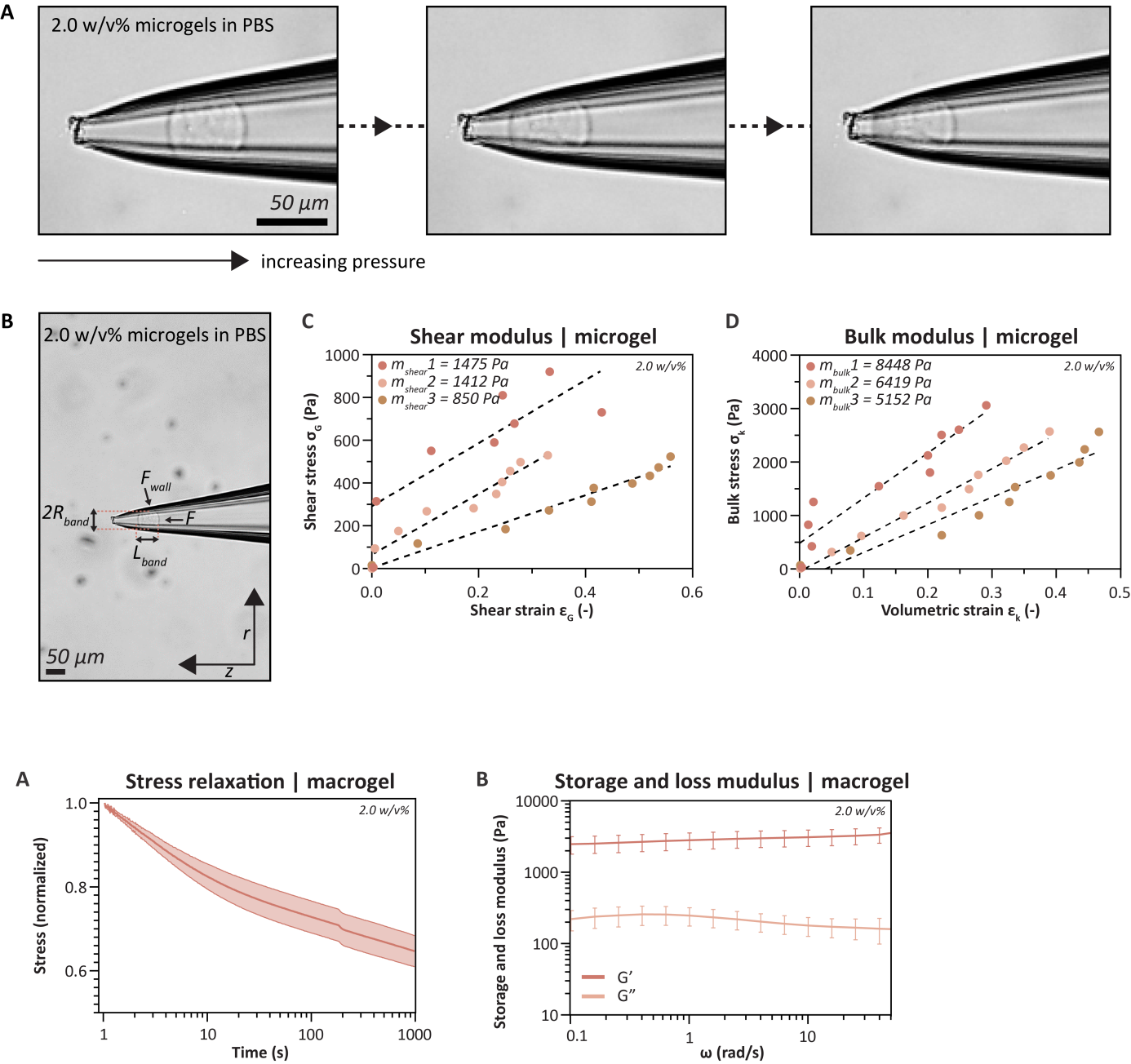


**Figure S3:** **Mechanical properties of macrogels.** On a macroscopic level we used rheology on 2.0 w/v% macrogels (n = 3). **A)** Stress relaxation behavior of a 2.0 w/v% macrogel measured by subjecting the hydrogel to 1% strain. **B)** Frequency dependence of storage (G’) and loss (G”) moduli of the macrogel.


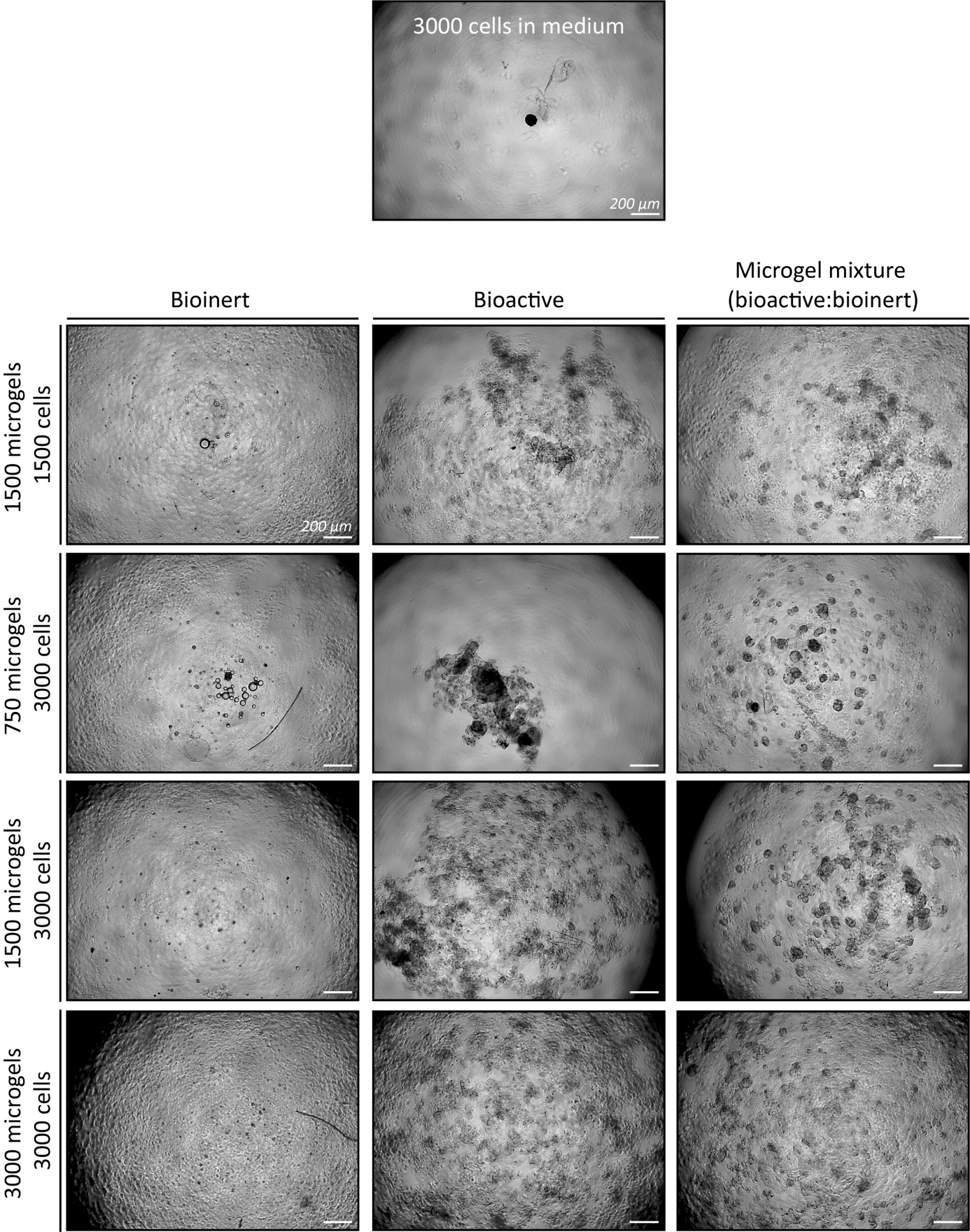


**Figure S4: Brightfield images of NHDF cultured on bioinert and bioactive microgels,** and on a mixture of both in a 1:1 ratio after 7 days of culture. Various microgel concentrations in combination with cell numbers were followed over time. NHDF cultured without microgels were used as a control. Scale bar represents for all images 200 μm.


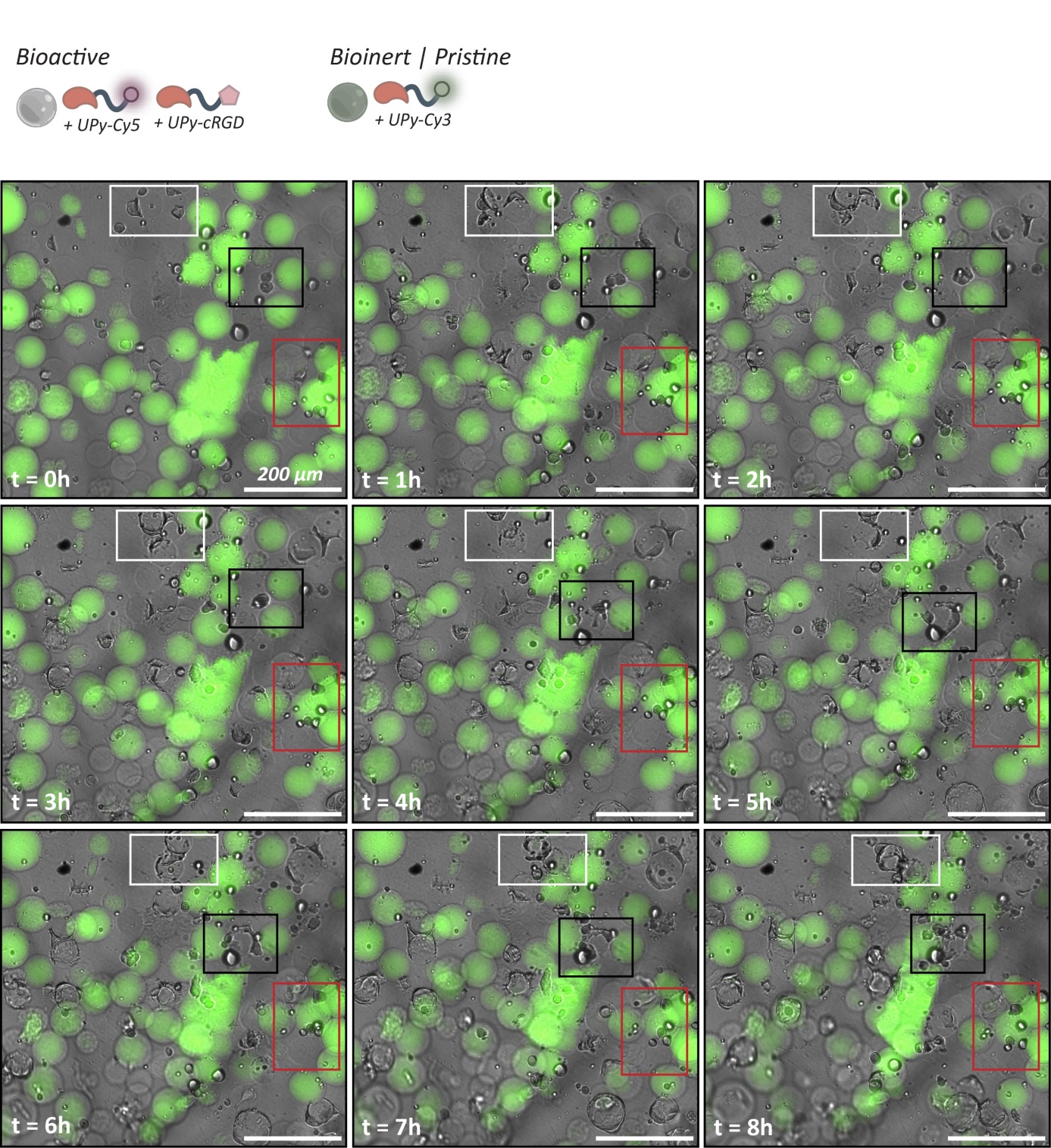


**Figure S5:** **Fluorescent time-lapse imaging.** NHDF were cultured on a mixture of bioinert (green) and bioactive (brightfield) microgels in the ratio 1:1 (3000 microgels with 3000 cells). The cells demonstrated the capacity to distinguish between the two microgel conditions by migrating towards the bioactive microgels when these were present in close proximity. Three distinct cell populations were identified: (1) cells adhering directly to a bioactive microgel and spreading (white box), (2) cells initially adhering to a bioinert microgel but capable of reaching a bioactive microgel and initiating spreading (black box), and (3) cells remaining rounded on the bioinert microgel (red box). All scale bars represent 200 μm.


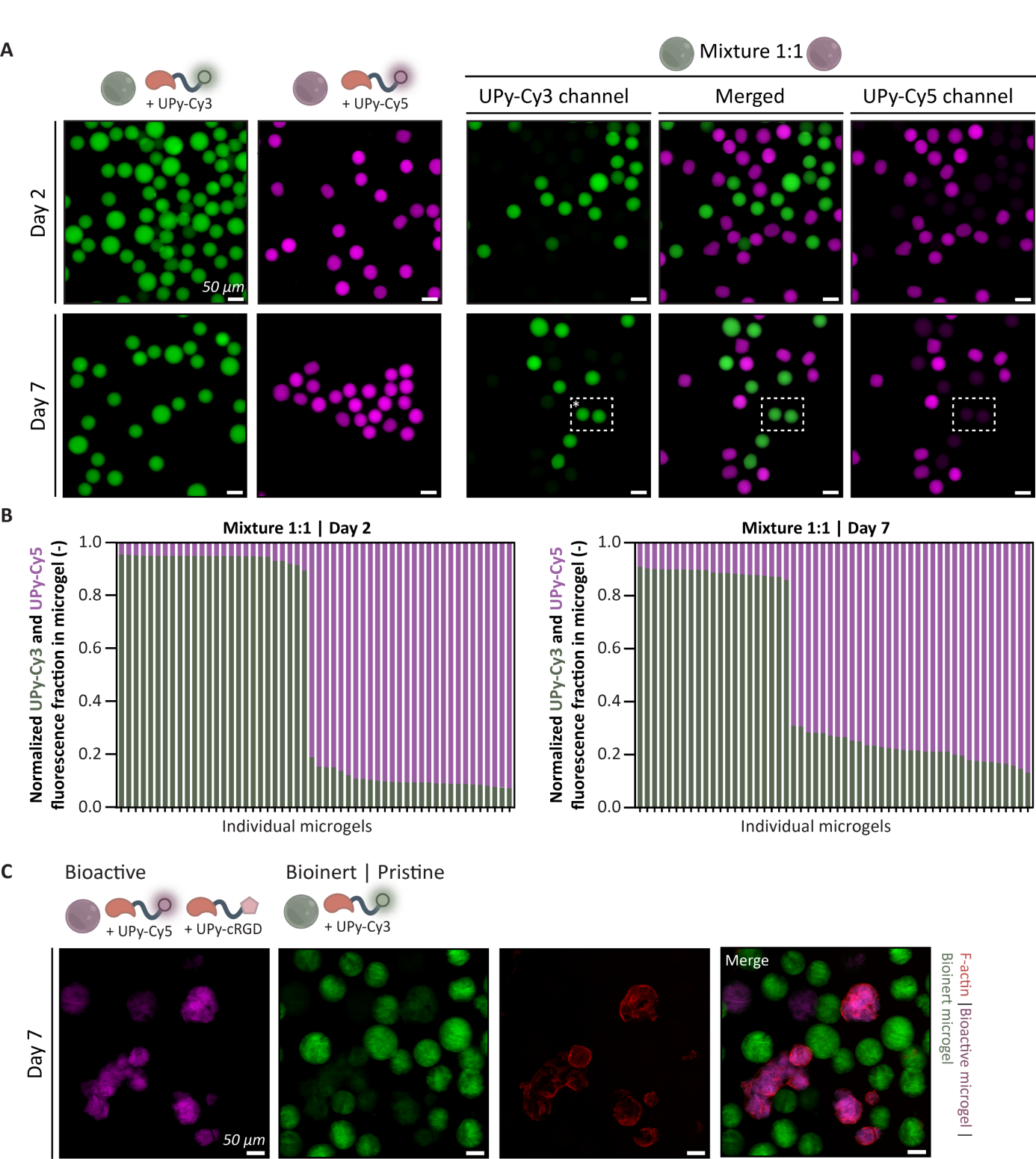


**Figure S6: UPy-dye exchange over period of 7 days. A)** Microgels of 2.0 w/v% were functionalized with a UPy-Cy3 or UPy-Cy5 dye and mixed together in a 1:1 ratio. Microscopy images reveal the exchange of UPy-Cy3 and UPy-Cy5 from one microgel to another after a period of 7 days (* indicated by the dotted square). **B)** The total fluorescence intensity of each microgel was measured and the portion corresponding to the UPy-Cy3 and UPy-Cy5 dyes was determined and plotted as a normalized fraction of UPy-Cy3 and UPy-Cy5 within each microgel. After 7 days, more UPy-dye was exchanged, as indicated by the increased normalized fraction compared to day 2, with UPy-Cy5 exchange rising from a maximum of 0.1 to 0.14, and UPy-Cy3 exchange increasing from a maximum of 0.19 to 0.31. **C)** NHDF cultured on a mixture of bioactive and bioinert microgels still showed solely cell adherence to the bioactive microgels, after 7 days of culture, despite the dye exchange. For all images: F-actin in red, and bioinert and bioactive microgels in green and magenta, respectively. All scale bars represent 50 μm.


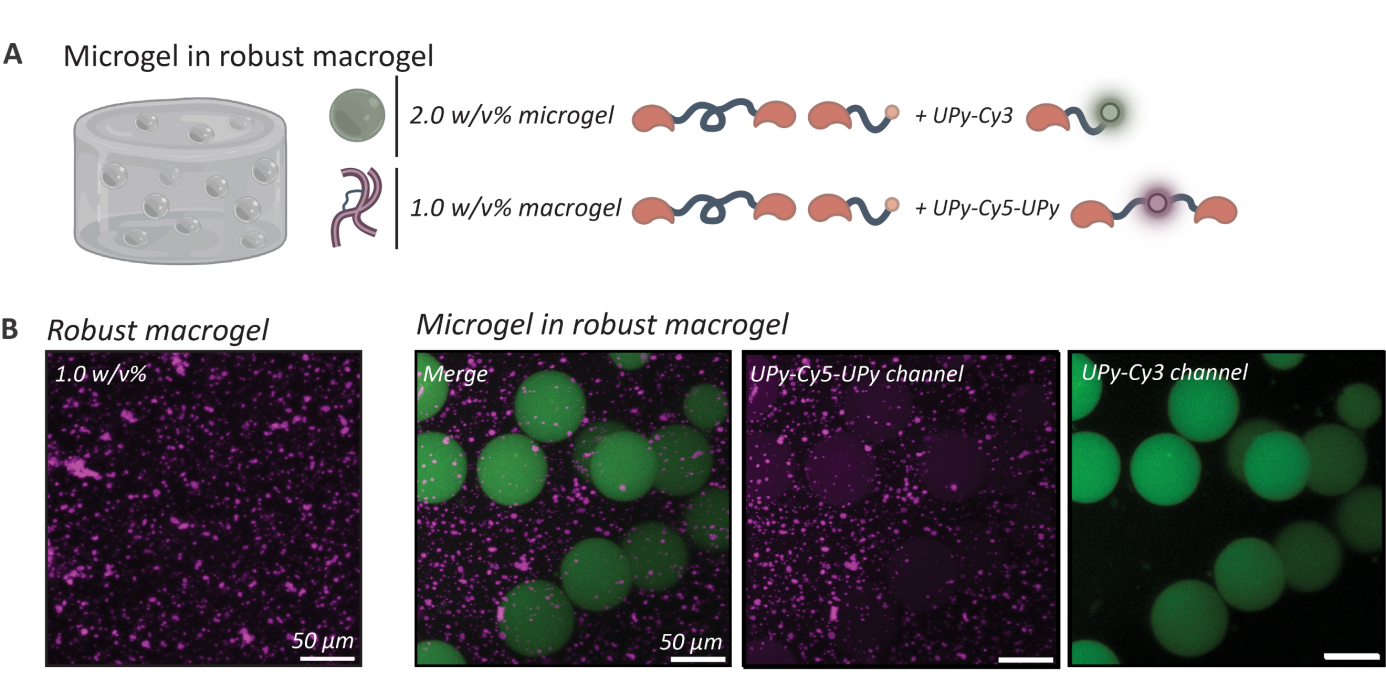


**Figure S7: Multicompartmentalized hydrogel by microgel in robust macrogel. A)** Schematic overview of a multicompartment hydrogel system established by the integration of 2.0 w/v% microgels (modified with UPy-Cy3) within a 1.0 w/% macrogel composed of B- and M-type molecules (modified with UPy-Cy5-UPy). **B)** Confocal images reveal the distinct microgel compartment (green) in the macrogel (magenta). Scale bars represent 50 μm.


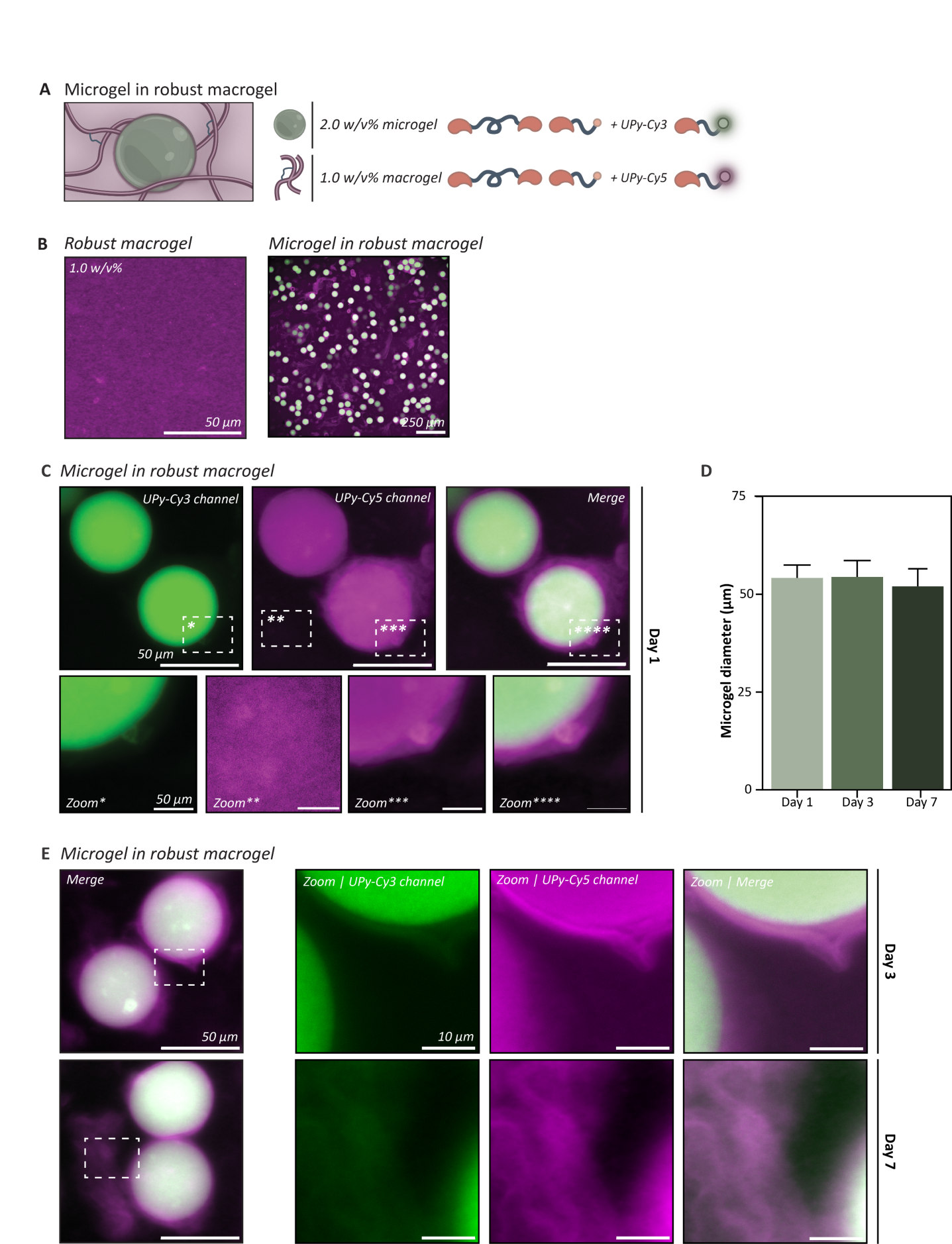


**Figure S8: Multicompartmentalized hydrogel by microgel in robust macrogel. A)** Schematic overview of a multicompartment hydrogel system established by the integration of 2.0 w/v% microgels (modified with UPy-Cy3; green) within a 1.0 w/% macrogel composed of B- and M-type molecules (modified with UPy-Cy5; magenta). **B)** Confocal images reveal the distinct microgel compartment (green) in the macrogel (magenta). Scale bars represent 50 and 250 μm. **C)** Zoomed-in images of the microgels in the macrogel. Scale bars represent 50 μm. **D)** The microgel diameter of encapsulated microgels in the bulk hydrogel does not change over a period of 3 and 7 days. **E)** At day 3, M-type molecules from the macrogel condensate on the microgel interphase (magenta), while there is little exchange of M-type molecules from the microgel (green) into the bulk. A similar effect was observed at day 7, however more exchange from the microgel, visible in green, was observed. Scale bars represent 50 μm and 10 μm for the zoomed-in images.
